# Identification of an enzyme with strong single-stranded DNA ligation activity and its application for sequencing

**DOI:** 10.1093/nar/gkaf054

**Published:** 2025-01-30

**Authors:** Fumihito Miura, Yukiko Shibata, Miki Miura, Kazune Inatomi, Yutaka Suzuki, Takashi Ito

**Affiliations:** Department of Biochemistry, Kyushu University Graduate School of Medical Sciences, Maidashi 3-1-1, Higashi-Ku, Fukuoka 812-8582, Japan; Life Science Data Research Center, Graduate School of Frontier Sciences, the University of Tokyo, Kashiwanoha 5-1-5, Kashiwa, Chiba277-8561, Japan; Department of Biochemistry, Kyushu University Graduate School of Medical Sciences, Maidashi 3-1-1, Higashi-Ku, Fukuoka 812-8582, Japan; Life Science Data Research Center, Graduate School of Frontier Sciences, the University of Tokyo, Kashiwanoha 5-1-5, Kashiwa, Chiba277-8561, Japan; Department of Biochemistry, Kyushu University Graduate School of Medical Sciences, Maidashi 3-1-1, Higashi-Ku, Fukuoka 812-8582, Japan; Life Science Data Research Center, Graduate School of Frontier Sciences, the University of Tokyo, Kashiwanoha 5-1-5, Kashiwa, Chiba277-8561, Japan; Department of Biochemistry, Kyushu University Graduate School of Medical Sciences, Maidashi 3-1-1, Higashi-Ku, Fukuoka 812-8582, Japan

## Abstract

An enzyme with strong single-stranded DNA (ssDNA) ligation activity would be advantageous for many molecular biology applications. However, currently available enzymes exhibit only limited activity. Here, we identified an enzyme with strong ssDNA ligation activity upon searching the databases for proteins homologous to TS2126 RNA ligase, the known enzyme with the highest yet limited ssDNA ligation activity. A ligase from Thermus phage phiLo (GenBank: AYJ73970) or Tph ssDNA ligase (SDL) depicted higher ssDNA ligation activity than TS2126 RNA ligase. This study suggests that SDL could be useful for various applications including sequencing for library preparation.

## Graphical Abstract

**Graphical Abstract:**
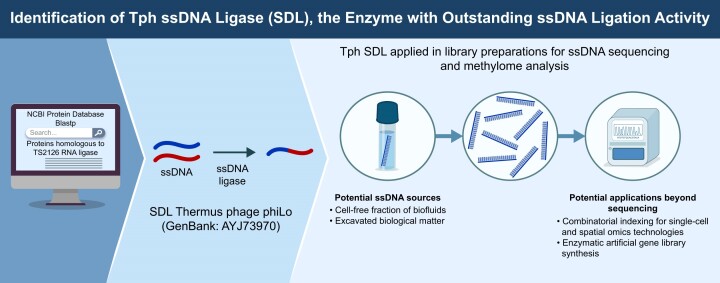

## Introduction

Although many applications are expected to be realized with an enzyme having efficient single-stranded DNA (ssDNA) ligation activity, no appropriate enzyme is currently available. The importance of an efficient ssDNA ligation technique is evident in sequencing library preparation. Many kinds of ssDNA have garnered interest for sequencing purposes. For example, short ssDNA in the cell-free fraction of blood, with characteristics different from those of nucleosome-sized cell-free DNA (cfDNA), has been recently identified [1–3]. DNA extracted from bones excavated from remains found several thousand years ago is usually heavily degraded, damaged, and single-stranded [4]. Further identification of potential ssDNAs requires sequencing. However, most commercially available library preparation kits are currently adapted for only double-stranded DNA (dsDNA). Although a limited number of methods are available for ssDNA, the library preparation efficiency of these ssDNA-adapted procedures is not sufficiently high [1, 5, 6].

To connect an adapter to ssDNA, one can use an enzyme with ssDNA ligation activity. T4 RNA ligase has long been known to have ssDNA ligation activity. Although the RNA ligase is highly active in ligating RNA, as its name suggests, its activity in joining two ssDNA molecules is minimal. Additives such as polyethylene glycol (PEG) and hexamine cobalt increase the ssDNA ligation activity of T4 RNA ligase [7]. However, even with these additives, the ssDNA ligation activity of T4 RNA ligase is practically low. Therefore, only applications based on polymerase chain reaction (PCR) amplification, such as rapid amplification of cDNA end, have been developed for ssDNA ligation activity of T4 RNA ligase [8, 9].

In the early 2000s, RM378 and TS2126 RNA ligases were identified from thermophilic viruses, which demonstrated superior ssDNA ligation activity than T4 RNA ligases [10, 11]. However, they had limited ssDNA ligation activity. Later, CircLigase II, which is an enzyme developed for ssDNA circularization, as the name indicates, was used to prepare the library from ssDNA [5]. However, its efficiency in library preparation from ssDNA was low or limited to only short ssDNAs. Indeed, the practical efficiency of CircLigase II is lower than that of random splint-based ssDNA ligation using T4 DNA ligase [6]. Such sequencing library preparation procedures using the random splint and T4 DNA ligase have been commercialized recently by Claret Bioscience.

The low ssDNA ligation activity of RNA ligases can be explained by the differential kinetic characteristics of the enzymes depending on ssDNA and RNA. RNA ligases catalyze three reactions [12, 13]. The first is the adenylation of enzymes themselves using adenosine triphosphate (ATP). Then, the adenyl moiety on the enzyme is transferred to the 5′-end of the 5′-phosphorylated oligo- or polynucleotide (donor) in the second reaction. Finally, RNA ligases connect the adenylate-activated donor to the 3′-hydroxyl end of oligo- or polynucleotide (acceptor). The existence of either DNA or RNA acceptors accelerates enzymes in the first and second reactions. When RNA is used as an acceptor, RNA ligases catalyze these three reactions in a well-balanced manner, i.e. the enzyme conducts all of the three reactions, completing the joining reaction. However, when DNA is used as an acceptor, RNA ligases primarily catalyze the first and second reactions, and most enzymes become adenylated. Because adenylated enzymes cannot execute the third reaction [12], the active enzyme without adenylation for the third reaction is depleted, causing inefficient ssDNA ligation.

To circumvent the lack of an efficient enzyme for ssDNA ligation, we recently invented terminal deoxyribonucleotidyl transferase (TdT)-assisted adenylate connector-mediated ssDNA (TACS) ligation [14]. In this method, the 3′-end of target ssDNA is first tailed with a few adenylic acids using TdT [15]. This short RNA stretch or ribotail is used as a connector for ligation with TS2126 RNA ligase [11]. Since the ribotailed ssDNA is a better acceptor for RNA ligases than the original ssDNA, the ligation efficiency is greatly improved. The efficiency of TACS ligation exceeds more than 80% at its optimum condition [14]. Therefore, TACS ligation can be used for highly efficient sequencing library preparations from cfDNA [1,16] and degraded DNA extracted from ancient human bones [16]. However, the ribotail could be an obstacle to developing new technologies that need to avoid insertions and RNA. Also, the connector RNA requires reverse transcriptase-like activity for DNA polymerase to replicate the product, meaning high-fidelity DNA polymerases with proofreading exonuclease activity cannot be used.

In the present study, we aimed to screen RNA ligases to identify new enzymes with ssDNA ligation activity without the help of ribotail, investigate the optimum reaction conditions for single-strand DNA ligase (SDL), and apply it for sequencing library preparations for both general ssDNA and methylome analysis.

## Material and methods

### Database search for RNA ligases

All nucleotide and amino acid sequence analysis was conducted with the CLC main workbench (Qiagen, Hilden, Germany). A BLASTP search was conducted with the amino acid sequence of TS2126 RNA ligase (UniProtKB accession number C0HM52) as a seed against the nr database at NCBI. Fifty sequences with the highest scores were selected, and multiple sequence alignment and phylogenetic tree analysis were performed. Amino acid sequences closely related to TS2126 RNA ligase were chosen and further analyzed (see below).

### Artificial gene synthesis, protein expression, and purification

The genes, namely encoding AYJ37970, RTT02525, WP_081 892 735, WP_110 532 308, WP_119 360 902, and WP_126 206 484, were synthesized by Twist Biosciences (South San Francisco, CA) with codon optimization for *E. coli* K-12 (Supplementary Information). These genes were PCR amplified and cloned into pET28a-StrepSL (Supplementary Information), and the correctness of insert sequences was confirmed with Sanger sequencing.

The cultivation of bacterial transformants, induction of protein expression, and purification of the recombinant proteins were conducted with a dual-affinity strategy comprised of 6X-His- and Strep-tag purifications described in our previous work for the purification of the TS2126 RNA ligase [1].

### Oligonucleotides

All the oligodeoxyribonucleotides (ODN) and oligoribonucleotides used in the present study were synthesized by Eurofin Genomics Japan (Tokyo, Japan), Integrated DNA Technologies Inc. (Commercial Park, IA), and FASMAC (Kanagawa, Japan). The sequences, modifications, and their purification grades are listed in Supplementary Table S1.

### Screening of ligases with ssDNA ligation activity

A 25 μL reaction containing 2 pmol acceptor (N100, Supplementary Table S1), 25 pmol of the donor (P-anti-PEA2-thio5-P, Supplementary Table S1), 400 μM ATP, 50 mM HEPES-KOH, pH7.5, 0.5% (v/v) Triton X-100, 20% (w/v) PEG #6000, and 2 μg of RNA ligase was prepared, mixed, and incubated sequentially for 30 min each at 37°C, 45°C, 55°C, and 65°C. If necessary, 10 units of TdT (Takara Bio Inc., Shiga, Japan) were added in the same reaction. After inactivating the enzyme with heating at 95°C for 5 min, 25 μL of water, 45 μL of buffer B2 (Qiagen), and 5 μL of proteinase K (Qiagen) were added and further incubated at 50°C for 15 min. The reaction solution was mixed with 100 μL of isopropanol and 2 μL of Sera-Mag Carboxylate Magnetic Beads (Cytiva, Marlborough, MA). After washing the magnetic beads with 70% (v/v) ethanol, the purified DNA was eluted with 10 μL 10 mM Tris-Acetate, pH 8.0, and analyzed on 10% Novex TBE-Urea Gels (Thermofisher Scientific) with SYBR Gold gel stain (Thermofisher Scientific).

### Optimization of reaction conditions of SDL

The detailed conditions used for the optimizations of the SDL reaction are described in the legend of individual figures. After optimization, we defined 1 × SDL buffer as comprising 50 mM Tris-HCl, pH 8.5, 0.05% (v/v) Triton X-100, and 10 mM MgCl_2_ (Supplementary Methods).

### Electrophoresis-based investigation of nucleotide preference of SDL

A 50 μL reaction solution containing 1 × SDL buffer, 30%(v/v) PEG #600, 200 μM ATP, 2 μM donor, 2 μM acceptor, and 10 μg (228 pmol) of SDL was prepared and incubated at 37°C. Then, the 2 μL of reaction solution was analyzed at 1, 2, and 4 h after starting the incubation. The solution was subsequently mixed with 10 μL of formamide, incubated at 70°C for 5 min, and analyzed on 10% Novex TBE-Urea Gels with SYBR Gold gel stain. The donor and acceptor used for individual reactions are indicated on the panel, and their sequences are listed in Supplementary Table S1.

### Sequencing-based investigation of nucleotide preference of SDL

#### Analysis of nucleotide bias at 5′-terminal side

A 25 μL reaction solution containing 1 × SDL buffer, 30%(v/v) PEG #600, 200 μM ATP, 1 μM PEA1T (Supplementary Table S1), 0.08 μM P-N40-anti-PEA2-NH2 (Supplementary Table S1), and 5 μg of SDL was prepared and incubated at 37°C for 1 or 8 h, followed by incubation at 95°C for 5 min.

#### Analysis of nucleotide bias at 3′-terminal side

A 25 μL reaction solution containing 1 × SDL buffer, 30%(v/v) PEG #600, 200 μM ATP, 1 μM PEA1T-N40 (Supplementary Table S1), 0.08 μM P-anti-PEA2-NH2 (Supplementary Table S1), and 5 μg SDL was prepared and incubated at 37°C for 1 or 8 h, followed by incubation at 95°C for 5 min.

#### DNA purification and indexing

The reaction after ligation was supplemented with 2 × KOD One (Toyobo, Tokyo, Japan) and 50 pmol PEA2 (Supplementary Table S1). Then, the reaction was incubated sequentially at 95°C for 3 min, 55°C for 5 min, and 72°C for 5 min. The product DNA was purified with the QIAQuick PCR purification kit (Qiagen) with an elution volume of 25 μL. The eluted DNA was supplemented with 2 × KOD One, 400 nM Primer-3, and 400 nM Index-X (X denotes the index number, Supplementary Table S1) and incubated at 95°C for 1 min, followed by ten cycles of three-step incubations of 15 s at 95°C, 55°C, and 72°C. The amplified and indexed library was purified using the QIAQuick PCR purification kit, and the concentration was determined using the Library Quantitation kit from Takara Bio Inc. Then, the purified PCR products with indexing were appropriately diluted and mixed for sequencing with iSeq 100 (Illumina, San Diego, California).

#### Calculating the degree of bias (DB)

The sequence reads were analyzed with a custom program named calc_bias to calculate nucleotide frequencies at each position of sequences. The mean nucleotide contents were calculated for 30 distal positions with respect to the end site of the ligation. The enrichment of the nucleobases for ten nucleotides proximal to the ligation site was calculated by normalizing these nucleobases with the mean compositions of 30 distal positions. The degree of bias (DB) was calculated by dividing the enrichment of the most enriched nucleotide with the least one. The results of the calculation were visualized using ggplot2 [17]. The codes for these calculations are provided in Supplementary Codes.

### Vaccinia virus topoisomerase I (VTopoI)-oligonucleotides complex (VOC) preparation

Vaccinia virus topoisomerase I (VTopoI) and T4 polynucleotide kinase (T4 PNK) were prepared as described previously [16].

A 1 mL reaction solution containing 1 × Oligo Hyb (Supplementary Methods) and 10 μM each of PEA1-3C2T-Ext, 1A3G1A-anti-PEA1, and Anti-Ext-1 (Supplementary Table S1) was prepared and sequentially incubated at 95°C for 3 min and 55°C for 5 min and finally maintained at 30°C. To the reaction solution, 2050 μL of water, 1000 μL 5 × TOPO activation buffer (Supplementary Methods), 500 μL of 1 mg/mL BSA (Takara Bio Inc.), 100 μL of ATP, and 100 μL of 150 μg/mL T4 PNK were added, followed by incubation at 30°C for 10 min. Then, 250 μL of 200 μM VTopoI was added, followed by incubation at 30°C for 1 h.

After the incubation, the reaction was loaded on a 1 mL HiTrap heparin column (Cytiva) pre-equilibrated with 5 mL of Buffer 300 (Supplementary Methods). After column washing with 5 mL of Buffer 300, the Vaccinia virus topoisomerase I (VTopoI)-oligonucleotides complex (VOC) was eluted with 5 mL of Buffer 400 (Supplementary Methods). The elution was loaded on an Amicon-4 30kDa device (Merck Millipore, Burlington, MA) to concentrate the VOC to 50 ng/μL. The concentration of VOC was determined by measuring the DNA concentration of the solution with the Qubit dsDNA BR kit (Thermo Fisher). This purification procedure was conducted with a syringe pump with a preset speed of 2 mL/min.

The VOC mentioned above was used for normal DNA library preparation (VOC for TA). For VOC for library preparation with bisulfite treatment, PEA1-1T-3C2T-Ext-met was used instead of PEA1-3C2T-Ext (VOC for BS).

### Purification of human blood cfDNA

The human serum was obtained from Kohjin Bio Co., Ltd (Saitama, Japan). The purification of cfDNA from 5 μL of the serum was performed using the PPAP method described previously [1].

### SDL-TOPO

#### First adaptor tagging

A 25 μL reaction solution containing 1 × SDL buffer, 400 μM ATP, 30% (v/v) PEG #600, 25 pmol P-AR-thio5-NH2 (Supplementary Table S1), sample DNA indicated, and 5 μg (114 pmol) of SDL was prepared and incubated sequentially at 37°C for 1 h and 95°C for 5 min.

#### Replication and purification

Subsequently, the solution was supplemented with 15 μL of water, 5 μL of 10 × PCR buffer (Takara Bio Inc.), 4 μL of 2.5 mM dNTPs, 1 μL of 50 μM NH2-AF (Supplementary Table S1), and five units of GeneTaq hot start (Nippon gene, Toyama, Japan) and incubated sequentially at 95°C for 3 min, 55°C for 5 min, and 72°C for 5 min. Then, 50 μL of AxyPrep Mag PCR clean and 50 μL of isopropanol were added and incubated at 5 min. The beads were washed twice with 200 μL of the solution containing 31% (v/v) PEG #400, 1 M NaCl, and 50 mM Tris-HCl, pH 8.0, and rinsed once with 70%(v/v) ethanol. The purified DNA was eluted with 25 μL of 10 mM Tris-Acetate, pH 8.0.

#### Second adaptor tagging and indexing

To the solution, 15 μL of water, 5 μL of 10 × ExTaq buffer, 4 μL of 2.5 mM dNTPs, 1 μL of ExTaq HS, 1 μL of 20 μM Primer-3, 1 μL of Index-X (X denotes an index number, Supplementary Table S1), and 1 μL of 5 ng/μl VOC for TA were added. After sequential incubation at 25°C for 5 min, 72°C for 5 min, and 95°C for 3 min, appropriate cycles of three-step incubations for 15 s at 95°C, 55°C, and 72°C, and a final extension at 72°C for 1 min were performed. Two cycles were used for library preparation without amplification.

### SDL-TOPO combined with bisulfite treatment

#### First adaptor tagging

A 25 μL reaction solution containing 1 × SDL buffer, 400 μM ATP, 30% (w/v) PEG #600, 25 pmol P-AR-Bs-thio5-NH2 (Supplementary Table S1), sample DNA indicated, and 5 μg of SDL was prepared and incubated sequentially at 37°C for 1 h and 95°C for 5 min.

#### Replication, second adaptor tagging, and DNA purification

Subsequently, the reaction solution was supplemented with 15 μL of water, 5 μL of 10 × PCR buffer, 4 μL of 2.5 mM dNTPs, 1 μL of 50 μM NH2-AF-Bs (Supplementary Table S1), and 5 units of GeneTaq hot start and incubated sequentially at 95°C for 3 min, 55°C for 5 min, and 72°C for 5 min. Then, 1 μL of 50 ng/μL VOC for Bs was added and incubated at ambient temperature for 5 min. The reaction was mixed with 250 μL of Buffer PB (Qiagen) and loaded on a ZymoSpin column (Zymo Research). After washing the column with 500 μL of Buffer PE (Qiagen), purified DNA was eluted with 20 μL of 10 mM Tris-HCl, pH 8.5.

#### Bisulfite treatment and PCR indexing

Purified DNA (20 μL) was used for treatment with the EZ DNA methylation gold kit (Zymo Research) according to the manufacturer's instruction (The final elution volume was 25 μL). After the bisulfite treatment, the DNA was supplemented with 25 μL of KOD One, 1 μL of 20 μM Primer-3 (Supplementary Table S1), and 1 μL of Index-X (X denotes the index number, Supplementary Table S1). After sequential incubation at 95°C for 3 min, appropriate cycles of three-step incubations of 15 s at 95°C, 55°C, and 72°C, and a final extension at 72°C for 1 min were performed.

### Comparison between tPBAT and sPBAT

Genomic DNA extracted from IMR90 cells and human female blood (Promega, Madison, WI) was used as a model for comparison of tPBAT and sPBAT protocols. Library preparations using the tPBAT protocol and genomic DNA extraction from IMR90 cells were performed as described previously [14]. For each preparation, 100 ng of genomic DNA was used.

### The sPBAT protocol

#### Bisulfite treatment, random priming, and DNA purification

Bisulfite treatment was performed using the EZ methylation gold kit with an elution volume of 40 μL. To the bisulfite-treated DNA, 5 μL of 10 × NEBuffer 2 (New England Biolabs, Ipswich, MA), 4 μL of 2.5 mM dNTPs, and 1 μL of 100 μL of PEA2-N4 (Supplementary Table S1) were added, followed by incubation at 95°C for 3 min and 4°C for 5 min. Then, 1 μL of 50 unit/μL Klenow fragment, exonuclease minus (New England Biolabs), was added to the reaction, followed by incubation at 4°C for 15 min. The reaction temperature was increased to 37°C at a rate of + 1°C/min, and the target temperature was maintained at 30 min. After heat-inactivation of the enzyme at 70°C for 10 min, the reaction solution was supplemented with 50 μL of AxyPrep Mag PCR clean and incubated at an ambient temperature for 5 min. After washing the beads twice with the 300-bp cutoff solution (Supplementary Methods) and once with 70% (v/v) ethanol, purified DNA was eluted with 12 μL of 10 mM Tris-Acetate, pH 8.0.

#### Second adaptor tagging with SDL

To the purified DNA, 5 μL of 10 × SDL buffer, 1 μL of 10 mM ATP, 1 μL of 50 μM PA-anti-PEA1-thio5-NH2 (Supplementary Table S1), and 15 μL of PEG #600 were added, followed by incubation at 95°C for 5 min and 37°C for 5 min. Then, 5 μg of SDL was added, and the reaction was incubated at 37°C for 1 h and 95°C for 5 min. To the reaction, 50 μL of AxyPrep Mag PCR clean was added, followed by incubation at an ambient temperature for 5 min. After rinsing the beads with 70% (v/v) ethanol, the purified DNA was eluted with 40 μL of 10 mM Tris-Acetate, pH 8.0.

#### Indexing and library QC

To the purified DNA, 5 μL of 10 × PCR buffer, 4 μL of 2.5 mM dNTPs, 1 μL each of Primer-3 and Index-X, and 1 μL of ExTaq HS (Takara Bio Inc.), were added, followed by incubation at 95°C for 3 min. Then, two cycles of three-step incubations at 95°C for 1 min, 55°C for 1 min, and 72°C for 1 min were performed. To the reaction, 20 μL of buffer B2 and 70 μL of AxyPrep Mag PCR clean were added, and the reaction solution was incubated at an ambient temperature for 5 min. The beads were washed twice with 400-bp cutoff solution (Supplementary Methods) and once with 70% (v/v) ethanol. The purified DNA was eluted with 20 μL of 10 mM Tris-Acetate, pH 8.0, and its concentration was determined using the Library Quantitation kit (Takara Bio Inc). The sequencing was performed with the NovaSeq X system at Macrojen Japan Inc.

## Results and discussion

### Identification of an enzyme with strong ssDNA ligation activity

**Figure 1.**
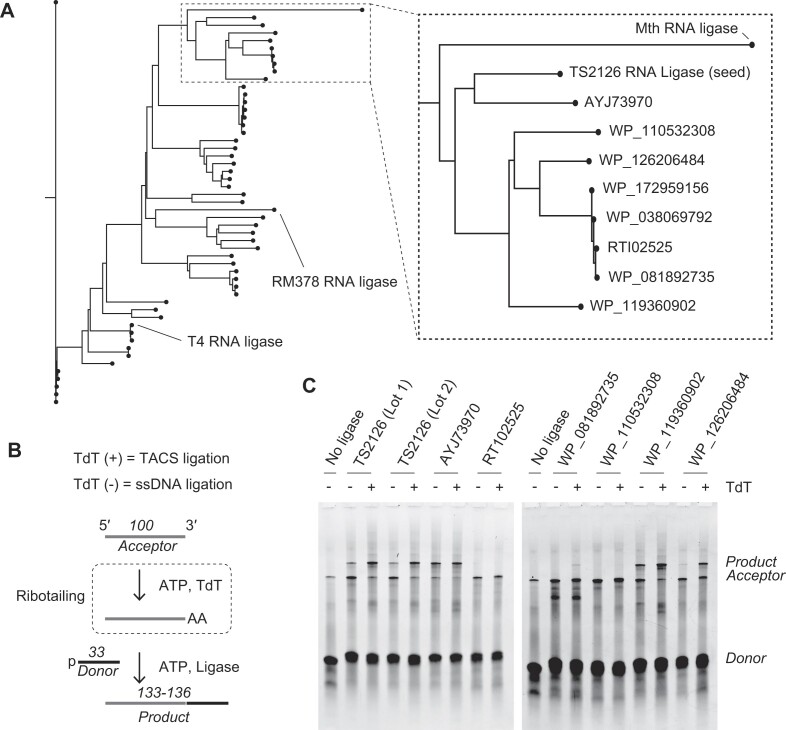
Identification of ssDNA ligase. (**A**). Phylogenetic tree of proteins homologous to TS2126 RNA ligase. The view reflected in the dashed box is enlarged in the right panel. (**B**). The assay design. TdT in the reaction attaches a few adenylates to the 3′-end of the acceptor to enhance the ligation activity of ligases (i.e. TACS ligation). In the absence of TdT, genuine ssDNA ligation activity could be measured. For the details, see the “Material and methods” section. (**C**). The gel images depict the results of assays. Successful ligation is indicated by a shift of the band toward the higher regions of the gel.

To identify enzymes with more robust ssDNA ligation activity, we BLAST-searched the NCBI protein database with TS2126 RNA ligase as the query protein sequence and found many homologous proteins. These involved several proteins with known ssDNA ligation activities, such as T4 RNA ligase [7, 18], RM378 RNA ligase [10], and Mth RNA ligase [19] (Fig. 1A). Interestingly, the phylogenetic tree indicated that these proteins of known ssDNA ligation activities were somewhat distant from TS2126 RNA ligase. Instead, eight proteins showed closer homologies to TS2126 RNA ligase (Fig. 1A). Various genome sequencing projects with different purposes have identified these eight proteins, but their ssDNA ligase activities have not yet been examined. Therefore, we decided to investigate the ssDNA ligation activities of these proteins. Of the eight proteins, two pairs have identical amino acid sequences but differ in N-terminal extension (RT102525 and WP_172 959 156; WP_081 892 735 and WP_038 069 792; Supplementary Fig. S1). From these four, we chose only the ones with N-terminal extensions (RT102525 and WP_081 892 735).

The six genes encoding these candidate enzymes (*AYJ73970, RT102525, WP_081 892 735, WP_110 532 308, WP_119 360 902*, and *WP_126 206 484*) were synthesized, cloned into an expression vector, and the encoded proteins were purified to near homogeneity (Supplementary Fig. S2). Further, the activities of these enzymes for both the TACS ligation (with ribotail) and ssDNA ligation (without ribotail) were analyzed (Fig. 1B). In this assay, we used randomized 100-mer (N100) for the acceptor and a sequencing adaptor for the donor. We found that three of the six proteins showed strong activity for TACS ligation (AYJ73970, WP_119 360 902, and WP_126 206 484, Fig. 1C). Two showed stronger ssDNA ligation activity than TS2126 RNA ligase (AYJ73970 and WP_119 360 902, Fig. 1C). Of these two, AYJ73970 showed more excellent ssDNA ligation activity (Fig. 1C). Hence, we investigated the use of AYJ73970 further.

### Optimization of reaction conditions for AYJ73930

AYJ73970 was active at 20–49°C with an optimum activity at 37°C (Supplementary Fig. S3). The coexistence of PEG with various molecular weights was required for its ligation activity (Supplementary Fig. S4). The solution pH should range between 7.5 and 9.0, and both Tris and TAPS-based buffer systems were preferable (Supplementary Fig. S5). Notably, in this pH range, acidic conditions are favorable for adenylation of donors, whereas alkaline conditions are preferable for ssDNA ligation (Supplementary Fig. S5). AYJ73970 lost its activity upon coexistence with ammonium chloride, even at 10 mM. In contrast, it showed activity with 50 mM sodium chloride or potassium chloride, with a preference for lower concentrations (Supplementary Fig. S6). The optimum magnesium concentration was 7.5–12.5 mM (Supplementary Fig. S7). For better ligation activity, ssDNAs with 16 and 30 nt in length were required for the donor and acceptor, respectively (Supplementary Fig. S8). While AYJ73970 showed no strong preferences between DNA and RNA acceptors, it preferred DNA over RNA as the donor (Supplementary Fig. S9). AYJ73970 was capable of ligating an ssDNA adaptor to dsDNA, albeit with significantly lower efficiency compared to ssDNA (Supplementary Fig. S10). AYJ73970 was found in the genome sequence of the bacteriophage Thermus phage phiLo. Therefore, it should be referred to as Tph ssDNA ligase; however, we have used the enzyme SDL for simplicity.

### Less 5′-adenylation proficiency may explain the good ssDNA ligation activity of SDL

**Figure 2.**
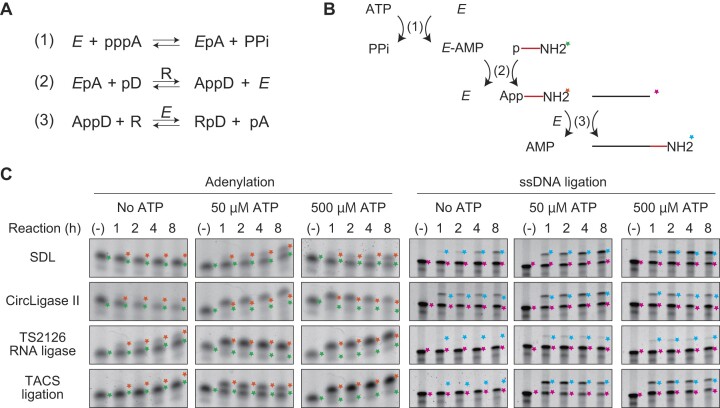
Comparisons of adenylation and ssDNA ligation activities of RNA ligases. (**A**). The proposed three reactions catalyzed by RNA ligases in literature are shown. (**B**). Schematics of the three reactions in A. In reaction (1), adenyl moiety is transferred from ATP to ligase. Then, the adenyl moiety is transferred to the 5′-phosphoryl moiety of donor DNA to produce an adenylated donor in reaction (2). Finally, the ligase connects the adenylated donor to the 3′-end of the acceptor to complete the ligation. The adenyl moiety on the adenylated donor is released as an adenosine monophosphate (AMP). (**C**). The adenylation and ssDNA ligation with SDL, CircLigase II, TS2126 RNA ligase, and TACS ligation using TS2126 RNA ligase are compared with three ATP concentrations (0, 50, and 500 μM). The colored stars on the right of each band indicate the substrates and products of adenylation and ssDNA ligation shown in (**B**). Terminal deoxynucleotidyl transferase (TdT) is included in TACS ligation. For the details, see Supplemental Methods. The symbols used in A and B are as follows. *E*: enzyme; pppA: ATP; *E*pA: enzyme-AMP complex; PPi: pyrophosphate; pD: 5′-phosphorylated donor; AppD: 5′-adenylated donor; R: acceptor (receptor); RpD: product; pA: AMP. The colors of stars in B and C are as follows: Green: 5′-phosphorylated donor; orange: adenylated donor; pink: unreacted acceptor; cyan: the ligation product.

Next, we focused on the differences of SDL with other RNA ligases in ssDNA ligation. We compared SDL with CircLigase II, TS2126 RNA ligase, and TACS ligation based on TS2126 RNA ligase for their products relating to reaction time and ATP concentrations. Two significant differences in the products of these ligases were evident. The first was the amount of 5′-adenylated donor produced. CircLigase II and TS2126 RNA ligase adenylated most donors within an hour in coexistence with ATP (Fig. 2). In contrast, only limited adenylation was observed with SDL after an 8-h reaction (Fig. 2). The second difference between SDL and other RNA ligases was the time-dependent accumulation of the ligation product. While increasing amounts of ligation product were observed upon prolonged incubation with SDL, only limited time dependency was observed with respect to the amount of ligation product obtained with CircLigase II, TS2126 RNA ligase, and TACS ligation (Fig. 2). These differences align with the previously discussed explanation for the low efficiencies of RNA ligases in ssDNA ligation.

The conversion rate of ligases to the self-adenylated form is dependent on the ATP concentrations (Fig. 2A and B). At the initial phase of the reaction, plenty of non-adenylated ligase molecules exist. At high ATP concentrations, self-adenylation proceeds more quickly to inactivate ssDNA joining activity, and adenylated donor molecules accumulate faster. In contrast, at low ATP concentrations, since the self-adenylation and production of adenylated donors is slow, active ligase exists in the early phase of the reaction. Therefore, the ligation reaction would take place more in lower ATP concentrations. Indeed, both CircLigase II and TS2126 RNA ligase showed enhanced ssDNA ligation activities under low ATP concentrations (50 μM, Fig. 2C). However, even in such low ATP concentrations, self-adenylation proceeds, and the transfer of adenyl moiety to the 5′-phosphorylated donor is completed in an hour (Fig. 2C). Under the coexistence of ATP, most adenylation-proficient ligases cannot avoid self-adenylation. The only way to exit the ligation-deficient adenylated form is by transferring the adenyl moiety to the 5′-end of the phosphorylated donor. However, the 5′-phosphorylated donor had already been used up after an hour of incubation. This could explain why CircLigase II and TS2126 RNA ligase showed limited time-dependency in accumulated ligation products.

In contrast, SDL showed less dependency on the ATP concentration for ssDNA ligation and produced minute amounts of adenylated product even with high ATP concentrations (500 μM, Fig. 2C). Moreover, SDL showed a time-dependent accumulation of ligation products with any ATP concentration (Fig. 2C). These observations indicate that low adenylation proficiency contributes to the high ssDNA ligation activity of the SDL.

### Nucleotide preference of SDL

**Figure 3.**
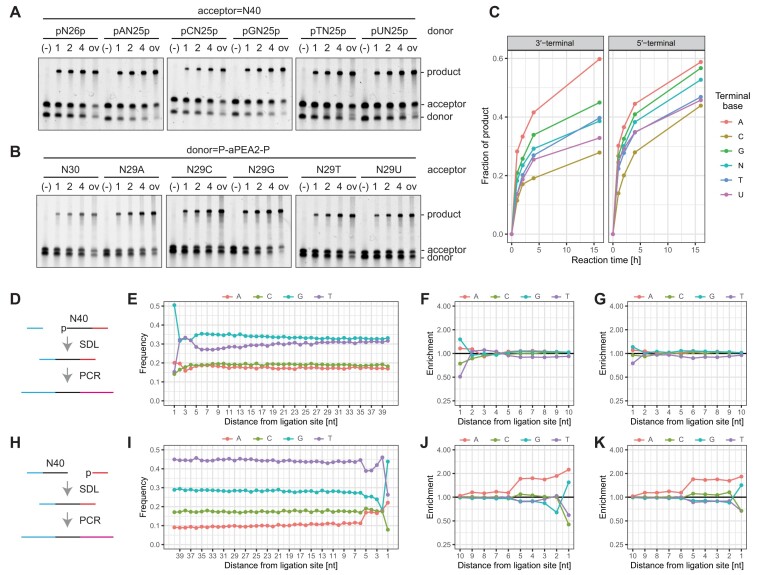
Nucleotide preferences of SDL A–C Electrophoresis-based analysis of nucleotide-dependent ligation efficiencies of SDL. The effects of 5′- (**A**) and 3′-terminal (**B**) nucleotides of the donor and acceptor, respectively, were analyzed with denaturing gel electrophoresis. (**C**). The relative signal intensities of the gels (**A and B**) are shown in the left and right panels, respectively. D–K Sequencing-based analysis of nucleotide-dependent ligation bias of SDL. (**D** and **H**) Schemes of template preparation used. E and I The examples of nucleotide compositions of sequenced reads. F, G, J, and K Normalized enrichment of nucleotide compositions of sequenced reads. The enrichments of nucleotides at 5′- (**F** and **G**) and 3′-terminal (**J** and **K**). Ten nucleotides of donor and acceptors, respectively, are shown. The durations of incubation at 37°C were 1 h (**F** and **J**) and 8 h (**G** and **K**).

RNA ligases show different activities dependent on the 5′- and 3′-terminal nucleotides of donor and acceptor molecules, respectively [20–24]. Therefore, we next investigated the nucleotide preference of SDL with electrophoresis of the ligation products. When assayed with ssDNA donors and acceptors with a specific nucleotide at their 5′ and 3′ ends, respectively, SDL showed stronger preferences with purines (A and G). In contrast, pyrimidines (C, T, and U) showed weak ssDNA ligation activities (Fig. 3A–C). We also investigated the effects of non-canonical nucleotides inosine (I) and apurinic/apyrimidinic (AP) site analog dSpacer (dSp) at the 5′-end of the donor on the SDL. We observed that SDL could connect ssDNA with these non-canonical nucleotides with different efficiencies (Supplementary Fig. S11). As the 5′-terminal nucleobase becomes smaller, the SDL might show reduced activity.

Nucleotide preferences of SDL would cause sampling biases in sequencing library preparation. We thus analyzed nucleotide compositions of libraries prepared from chemically synthesized N40 to examine how terminal nucleotide-dependent sampling bias affects the sequencing library preparation (Fig. 3E and I). Since differences in nucleotide balances at randomized sequences were observed across different batches of chemically synthesized ODN (Fig. 3E and I), we normalized the nucleotide enrichment at the proximal ten bases to the ligation site with the mean nucleotide composition of distal 30 bases (Fig. 3E–I).

We observed enrichments of purines at the 5′-most terminal base of donors with strong preferences for deoxyguanosine (dG, DB = 2.8 with SDL and 1-h incubation, Fig. 3F). Conversely, deoxyadenosines (dAs) were enriched on the 3′-terminal five bases of acceptors (DB = 6.8 with SDL and 1-h incubation, Fig. 3J). We also investigated the nucleotide preferences of TS2126 RNA ligase, CircLigase II, and TACS ligation in ssDNA ligation. We observed that such biases are commonly observed with these enzymes/reactions in ssDNA ligation (Supplementary Fig. S12A). Substantial preference was observed for the dG at the 5′-end of the donor with CircLigase II (DB = 15.6 with CircLigase II and 1-h incubation, Supplementary Fig. S12A). Similar preference was observed with SDL at increasing temperatures; however, the preference was less significant (DB = 3.4, 3.7, and 4.4 with incubation at 37, 42, and 47°C for 1 h, respectively, Supplementary Fig. S12A).

The nucleotide biases pose challenges in practical sequencing library preparation. Hence, we investigated ways to reduce such biases. Given that extended reaction times alleviated these nucleotide preferences (Fig. 3D–K), we hypothesized that further incubation would resolve the bias. As expected, prolonged incubation of up to 48 h improved the bias slightly (Supplementary Fig. S12B).

While prolonged incubation is effective, it compromises the practical usability of SDL for sequencing library preparation. Considering a general scheme for sequencing library preparation from ssDNA, the first step involves adaptor tagging to the 3′-end of target ssDNA with SDL (see below), and any defined sequence can be chosen for the adaptor. Because dG is highly preferable at the 5′-terminal of a donor (Fig. 3D–G), we hypothesized that placing a dG to the 5′-end of an adaptor might accelerate ssDNA ligation, thereby reducing the reaction time. As expected, the dG addition to an adaptor accelerated the ssDNA ligation and mitigated the nucleotide bias observed at the 3′-terminal five bases of an acceptor (DB = 2.1 with 1-h incubation, Supplementary Fig. S12C).

### General and efficient procedure for sequencing library preparation from ssDNA

**Figure 4.**
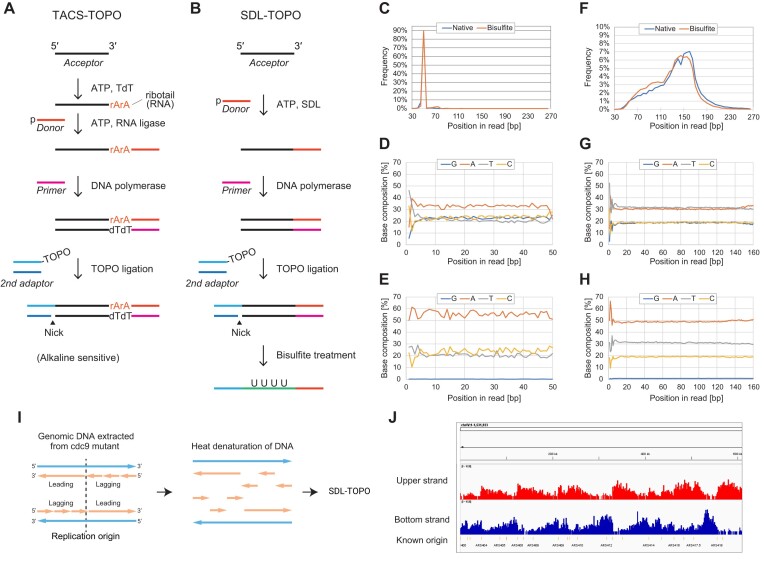
SDL-TOPO provides a general scheme for library preparation for genome and methylome analysis from ssDNA. (**A** and **B**). The schemes of library preparations with TACS-TOPO (**A**) and SDL-TOPO (**B**). In TACS-TOPO, the RNA residues in the library prevented alkaline treatment. In contrast, because the SDL-TOPO library did not comprise RNA, it can be served for bisulfite treatment. Library preparation with SDL-TOPO and sequencing results with and without bisulfite treatment on synthetic (**C**–**E**) and blood cfDNA (**F**–**H**) are shown. The length of inserts (**C** and **F**) and nucleotide compositions of reads without (**D** and **G**) and with (**E** and **H**) bisulfite treatment are shown. The frequencies of guanine in libraries with bisulfite treatment are quite low. Note that the complementary strand to the original DNA was determined in the sequencing. For the details, see Supplementary Methods. I and J. An application of SDL-TOPO for identification of replication origins. In ligase-deficient cells, *cdc9* mutants of the budding yeast *S. cerevisiae*, nicks and gaps are left unsealed after the genomic DNA replication. These short DNA fragments can be analyzed with SDL-TOPO after heat-denaturation of genomic DNA (**I**). Genome browser shot of an SDL-TOPO library prepared from genomic DNA extracted from a cdc9 mutant of yeast strain. The read depth mapped on the upper (red) and bottom (blue) strands is shown along with known replication origins (**J**). For details, see Supplementary Methods.

Having investigated the properties of SDL in detail, we next attempted to develop a general sequencing library preparation scheme using the SDL. We previously established a TACS ligation-based general sequencing library preparation procedure from ssDNA called TACS-TOPO [16]. The TACS-TOPO realized a highly sensitive analysis of short ssDNA in the cell-free fraction of human blood [16]. Furthermore, the TACS-TOPO method significantly increased yields in sequencing library preparations from ancient human bones by an order of magnitude [16]. Based on these past developments, we attempted to substitute the TACS ligation of the TACS-TOPO (Fig. 4A) with SDL (SDL-TOPO, Fig. 4B).

The library yields of both procedures, i.e. SDL-TOPO and TACS-TOPO, were almost the same; however, the latter performed slightly better when starting with a chemically synthesized random 50-mer DNA (N50) (Supplementary Fig. S13). The yields of SDL-TOPO were improved to the same level as those of TACS-TOPO when an adaptor with additional dG at the 5′-terminal end was used. This observation indicated that further analysis of nucleotide preferences and kinetics of SDL reaction would improve the ssDNA ligation efficiency. Accordingly, in terms of library preparation, SDL-TOPO demonstrates nearly the same efficiency as TACS-TOPO.

However, the SDL-TOPO exhibited an additional benefit compared to TACS-TOPO. Because the RNA is sensitive to alkaline conditions, and alkaline treatment is an indispensable step of bisulfite treatment, the product of the TACS-TOPO with ribotail could not be treated with sodium bisulfite (Fig. 4A). However, the library prepared with SDL-TOPO could be treated with bisulfite to investigate the 5-methylation status of insert DNA (Fig. 4B). As expected, bisulfite treatment eliminated cytosines in the SDL-TOPO library and increased the thymidines (Fig. 4C–H). Notably, 5mC-protected adaptors were used for bisulfite-treated library preparation to prevent the bisulfite-mediated conversion of adaptor sequences (see Methods section).

The size distributions of sequenced reads were almost the same with and without the bisulfite treatment (Fig. 4C and D), which indicates that SDL-TOPO with bisulfite reflected the compositions of cfDNA. Therefore, SDL-TOPO can be used for methylome analysis of blood cfDNA of both nucleosome-sized DNA and the recently identified short ssDNA.

Since SDL-TOPO has realized a highly efficient library preparation from ssDNA, we hypothesized that it could applied for detecting lagging strand DNA left unrepaired in budding yeast cells lacking nick-sealing DNA ligase *cdc9*. Previous methods for detecting such fragments required a microgram amount of genomic DNA and time-consuming procedures [25–29]. In contrast, the SDL-TOPO is highly efficient, and it only requires its target DNA to be single-stranded, which could be achieved with heat denaturation of extracted genomic DNA (Fig. 4I). Indeed, library preparation was successfully done with a sub-nanogram amount of genomic DNA (Supplementary Fig. S14). The read depth calculated with mapped reads on the reference genome showed typical strand-biased patterns of lagging strand sequencing (Fig. 4J and Supplementary Fig. S14). These results indicated that SDL-TOPO would provide simple and sensitive approaches to ssDNA-related biological questions.

### SDL improves the yields of random priming-based post-bisulfite adaptor tagging (PBAT) protocol

**Figure 5.**
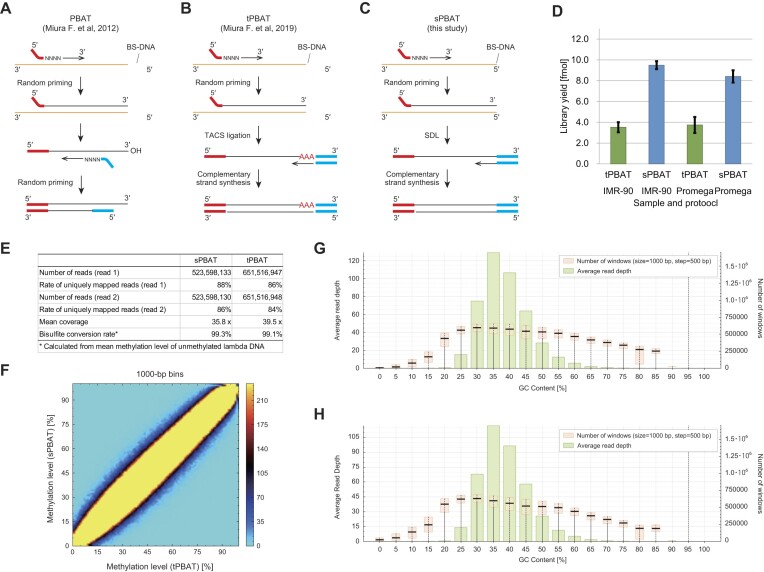
SDL Improves the yields of methylome library preparation with post-bisulfite adaptor tagging strategy. A–C. Schemes showing principles of post-bisulfite adaptor tagging protocols. The original PBAT protocol employed two successive random priming steps (**A**). However, repeating random primings led to the short insert of the library. Hence, the second random priming step was replaced with TACS ligation in tPBAT protocol (**B**). The TACS ligation was further replaced with SDL (**C**). D. The library yields between tPBAT and sPBAT were compared with two model DNA samples. E. The basic statistics of sequencing results on the IMR-90 libraries in D. F. The comparisons of IMR-90 methylomes measured by tPBAT and sPBAT. The mean methylation levels of CpGs in 1000-bp bins were compared. G and H. GC content-dependent sequencing bias were compared between tPBAT (**G**) and sPBAT (**H**).

For the quantification of genome-wide 5-methylation states of cytosines, whole-genome bisulfite sequencing (WGBS) is one of the practical options. Because the bisulfite treatment of the adaptor-tagged sequencing library sometimes leads to severe loss of the library, adaptor tagging after the bisulfite treatment (PBAT strategy) is effective for the library preparation of WGBS [30]. We have established protocols for the PBAT strategy. Two random priming reactions were sequentially conducted in the original version of the PBAT protocol (Fig. 5A) [30]. However, repeated random priming reactions shortened the library insert. Hence, instead of the second random priming reaction, we performed TACS ligation to establish tPBAT [14]. In the tPBAT protocol, bisulfite-treated DNA was replicated with random priming for the first adaptor tagging. Then, the TACS ligation was conducted for tagging the second adaptor to the first-strand DNA (Fig. 5B). TACS ligation relies on two enzymes, whereas SDL functions independently. Therefore, we hypothesized that substituting TACS ligation with SDL would simplify the WGBS library preparation and establish the sPBAT protocol (Fig. 5C). Because the ssDNA ligation efficiency of SDL was comparable to that of TACS ligation, we expected almost the same library yields with sPBAT and tPBAT.

Contrary to this expectation, sPBAT generated two to three times more library yields than tPBAT; however, the underlying reasons remain unclear (Fig. 5D). The quality of reads obtained with sPBAT was similar to that of tPBAT, with the same result on quantitation of DNA methylation levels (Fig. 5E–H). The operation of the sPBAT protocol is more simplified than that of tPBAT, which, in turn, enhances the practicality of methylome data production.

## Conclusions

In this study, we discovered an enzyme, SDL, with higher ssDNA ligase activity than other known ligases. Upon a detailed characterization of SDL, we observed a lower adenylation proficiency, which could contribute to its superiority in terms of ssDNA ligation. Similar to other RNA ligases, SDL showed terminal nucleotide-dependent ligation bias, which could distort sequencing results. However, this study showed that modifications of the adaptor would lower the deviation.

The use of SDL has been demonstrated for sequencing library preparations for general purposes and methylome analysis; however, these examples illustrate only a fraction of its potential applications. SDL offers opportunities for various novel applications. For example, SDL would be useful for combinatorial indexing, which has become a vital process for single-cell and spacial omics technologies. By mixing target DNA and 5′-phosphorylated ODN with SDL, effective indexing could be achieved. Similarly, enzymatic artificial gene library synthesis could be practical with SDL. Incorporating randomized codons and module shuffling of amino acid sequences, which was challenging with chemical synthesis, might be realized with SDL. SDL holds promise for numerous other applications beyond those discussed here.

While SDL showed a highly efficient ligation of ssDNA, it still has limitations, such as nucleotide preferences. Analysis of SDL structurally and kinetically could further deepen our understanding of the ssDNA ligation and lead to the development of a bias-free ssDNA ligase. The use of such enzymes would pave the way for advancements in DNA manipulation and synthesis techniques.

## Supplementary Material

gkaf054_Supplemental_Files

## Data Availability

All sequencing data produced in this study were deposited to NCBI GEO with accession numbers GSE268627 and GSE286342, as well as to DDBJ DRA with accession numbers DRX557905-DRX557908.
