# Supplementary material for: Identification of an enzyme with strong single-stranded DNA ligation activity and its application for sequencing
Source: Nucleic Acids Res. 2025 Jan 30;53(3):gkaf054. doi: 10.1093/nar/gkaf054 (PMC11780859; doi:10.1093/nar/gkaf054)
Supplement: gkaf054_Supplemental_Files [file gkaf054_supplemental_files.zip › Miura_ssDNA-ligase_SupplementaryInformation_20250115_clean-2.pdf]

Supplementary Information

Identification of an enzyme with strong single-stranded DNA ligation activity and its application for sequencing

Fumihito Miura[1, 2,*], Yukiko Shibata[1], Miki Miura[2], Kazune Inatomi[1], Yutaka Suzuki[2], and Takashi Ito[1]

[1] Department of Biochemistry, Kyushu University Graduate School of Medical Sciences, 3-1-1 Maidashi, Higashi-Ku, Fukuoka 812-8582, Japan

[2] Life Science Data Research Center, Graduate School of Frontier Sciences, the University of Tokyo

* To whom correspondence should be addressed. +81-4-7136-4069; Email: fumihito@fmfmnet.org

Supplementary Figures

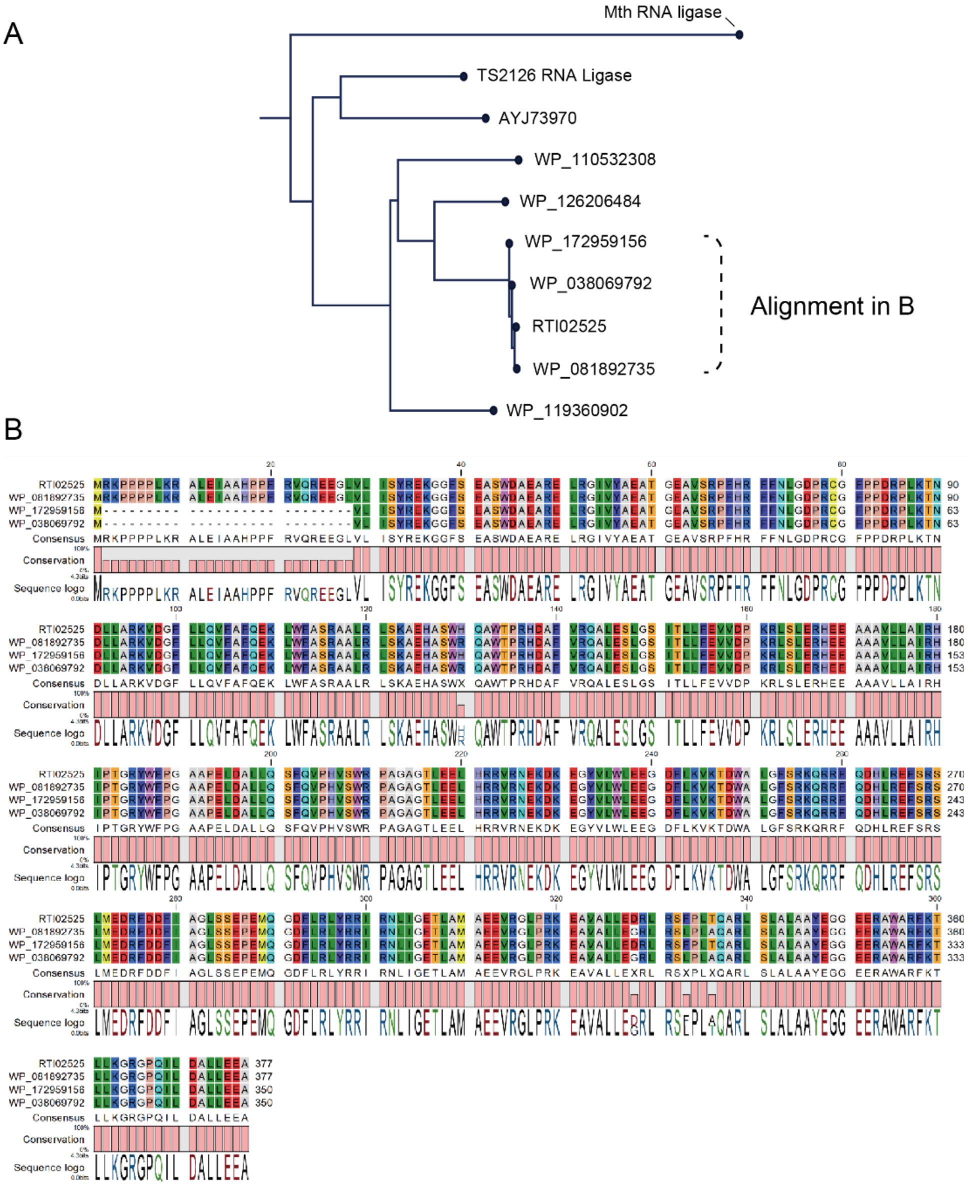

Supplementary Figure S1. Phylogenetic relationship among the candidate proteins. A. Partial phylogenetic tree of the amino acid sequences of proteins homologous to TS2126 RNA ligase. The same image of the enlarged part of Figure 1A is shown. B. Multiple sequence alignment of amino acid sequences indicated in A is shown. WP172959156 and RTI02525 exhibit the same amino acid sequence except for the N-terminal extension. The same is true for WP_081892735 and WP_172959156.

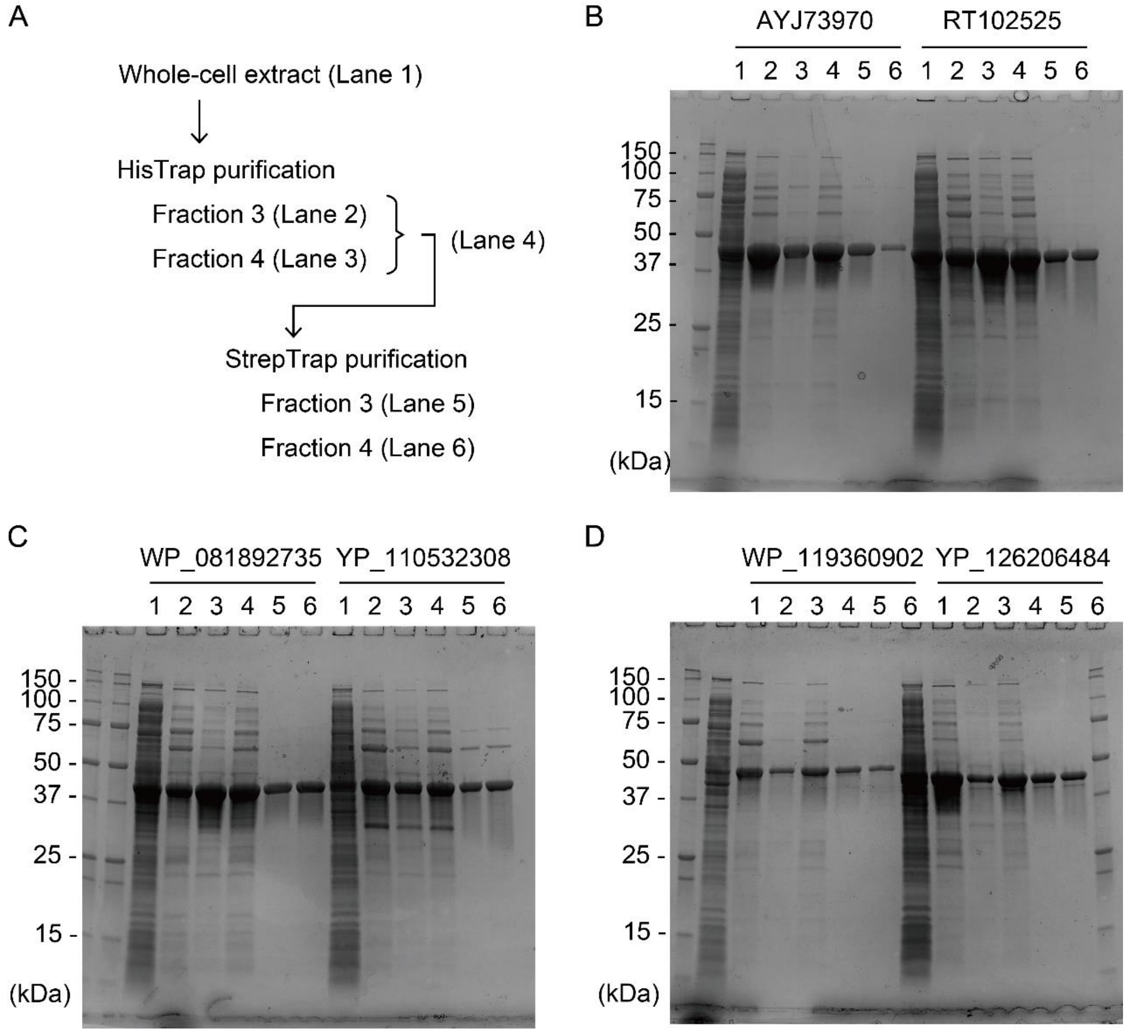

Supplementary Figure S2. Dual-affinity purification of six candidate proteins. A. The purification strategy. B–D. SDS-PAGE analysis of purified proteins. The legends of lane numbers are indicated in A. Lysates and purified proteins are loaded on mini protean TGX precast gels (Bio-rad laboratories, Hercules, CA). After electrophoresis, the gel was stained with Bullet CBB Stain One (Nacalai Tesque, Kyoto, Japan) and photographed with the ChemiDoc Touch imaging system (Bio-rad).

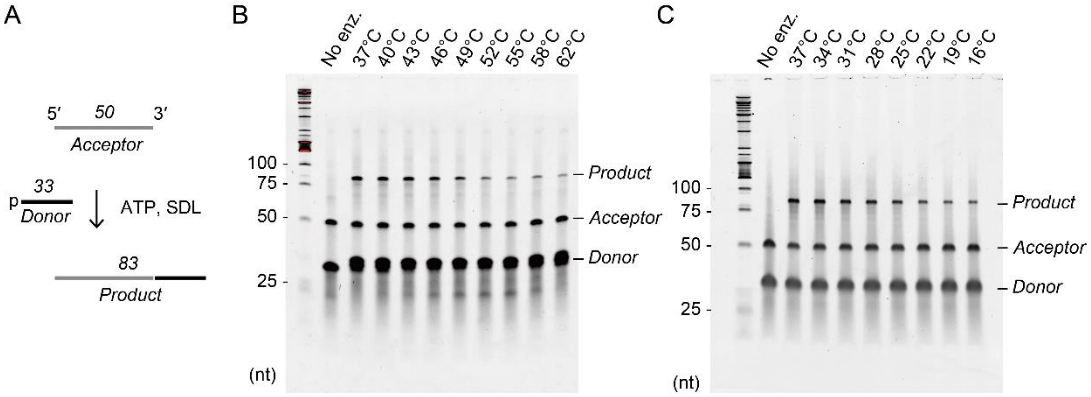

Supplementary Figure S3. The optimum reaction temperature for AYJ73970. A. The assay design. The numbers in italic fonts indicate the length of the oligonucleotides. B and C. Gel images depict the denaturing gel electrophoresis of the reaction. Reaction solutions containing either 50 mM HEPES-KOH, pH 7.5 (for B) or 50 mM Tris-HCl, pH 8.5 (for C), 10 mM $MgCl_2$, 0.05% (v/v) Tritton X-100, 400 μM ATP, 16% (w/v) polyethylene glycol (PEG) #6000, 5 pmol acceptor (N50, Supplementary Table S1), 50 pmol donor (P-anti-PEA2-thio5-P, Supplementary Table S1), and 2.8 μg (63 pmol) dual-affinity purified AYJ73970 (SDL) in 25 μL were prepared and incubated at the indicated temperature for 1 h. A reaction solution without ligase was prepared and incubated at 37°C for 1 h as a control. These solutions were supplemented with 20 μL Buffer B2 (Qiagen, Hilden, Germany) and 5 μL proteinase K (Qiagen) and incubated at 50°C for 15 min. Then, the solutions were supplemented with 2 μL of Sera-Mag carboxylate beads (Cytiva, Marlborough, MA) and 50 μL ethanol and incubated at room temperature for 5 min. After rinsing the beads with 200 μL of 70% ethanol, purified DNA was eluted with 10 mM Tris-Acetate, pH 8.0. An equivolume of purified DNA and formamide was mixed, heated at 70°C for 5 min, and loaded on 10% Novex TBE-Urea Gels (Thermofisher Scientific, Waltham, MA). After running, the gel was stained with SYBR Gold gel stain (Thermofisher Scientific) and photographed with the ChemiDoc Touch imaging system.

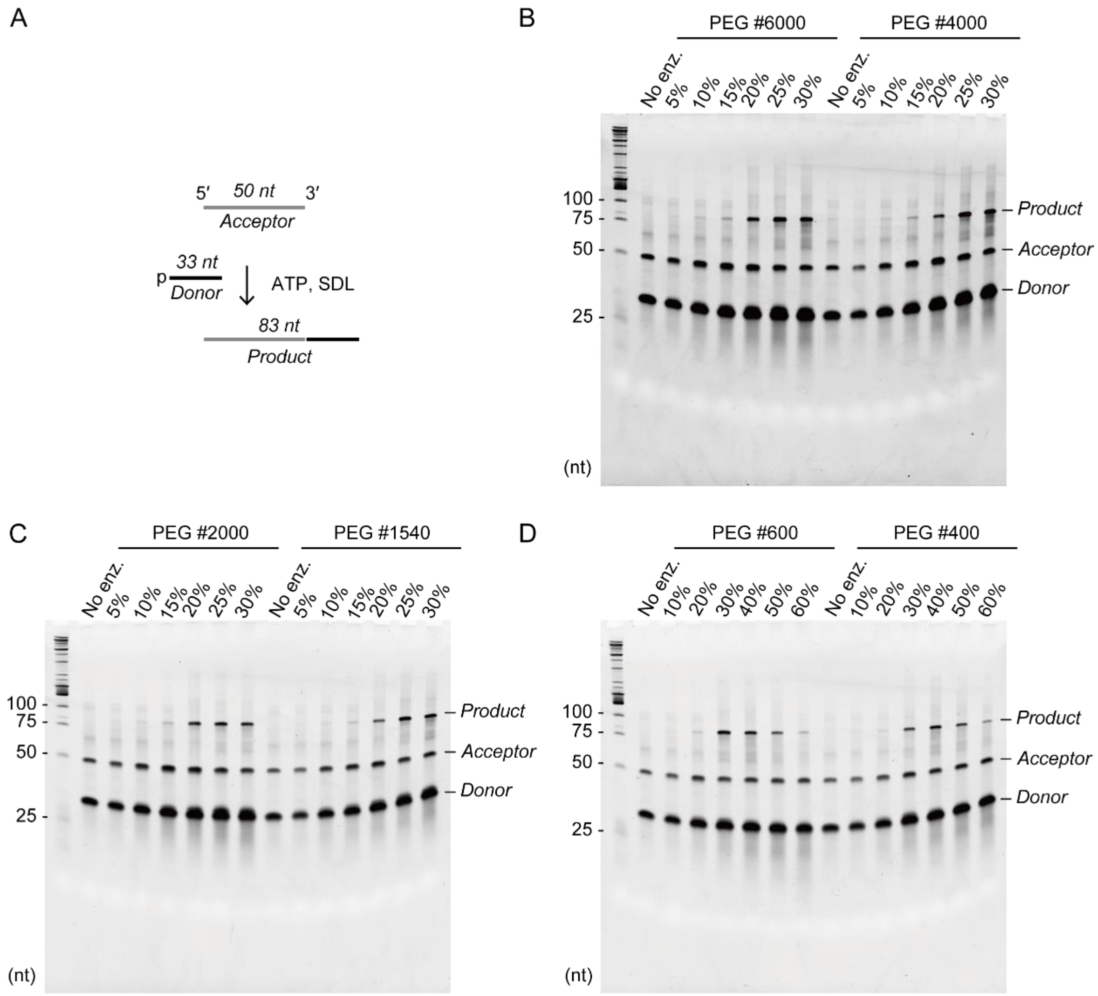

Supplementary Figure S4. The optimum concentrations of PEG of various molecular weights. A. The assay design. B–D. Gel image of the denaturing gel electrophoresis of the reaction. Reaction solutions containing 50 mM Tris-HCl pH8.5, 5 mM $MgCl_2$, 0.05% (v/v) Tritton X-100, 400 µM ATP, indicated PEG, 5 pmol acceptor (N50), 50 pmol donor (P-anti-PEA2-thio5-P) and 2.8 µg (63 pmol) dual-affinity purified AYJ73970 in 25 µL were prepared and incubated at 37°C for 1 h. As a control, a reaction solution without the ligase was prepared. The procedures used to purify the reaction and gel electrophoresis were the same as those in Supplementary Figure S3.

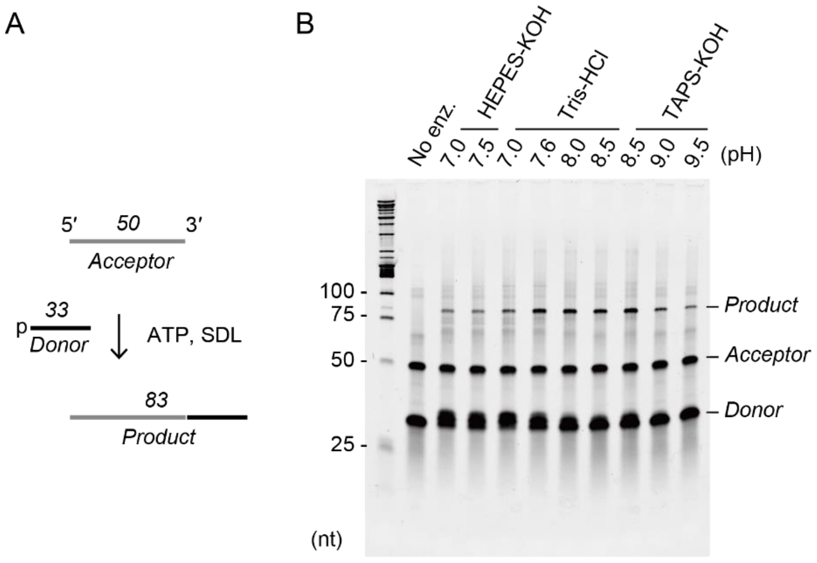

Supplementary Figure S5. The optimization of reaction pH and the buffering system. A. The assay design. B. Gel image of the denaturing gel electrophoresis of the reaction. Reaction solutions containing 50 mM buffer indicated, 5 mM $MgCl_2$, 0.05% (v/v) Tritton X-100, 400 μM ATP, 16% (w/v) PEG #6000, 5 pmol acceptor (N50), 50 pmol donor (P-anti-PEA2-thio5-P) and 2.8 μg (63 pmol) dual-affinity purified AYJ73970 in 25 μL were prepared and incubated at 37°C for 1 h. As a control, a reaction solution without ligase was prepared. The procedures used to purify the reaction and gel electrophoresis were the same as those in Supplementary Figure S3.

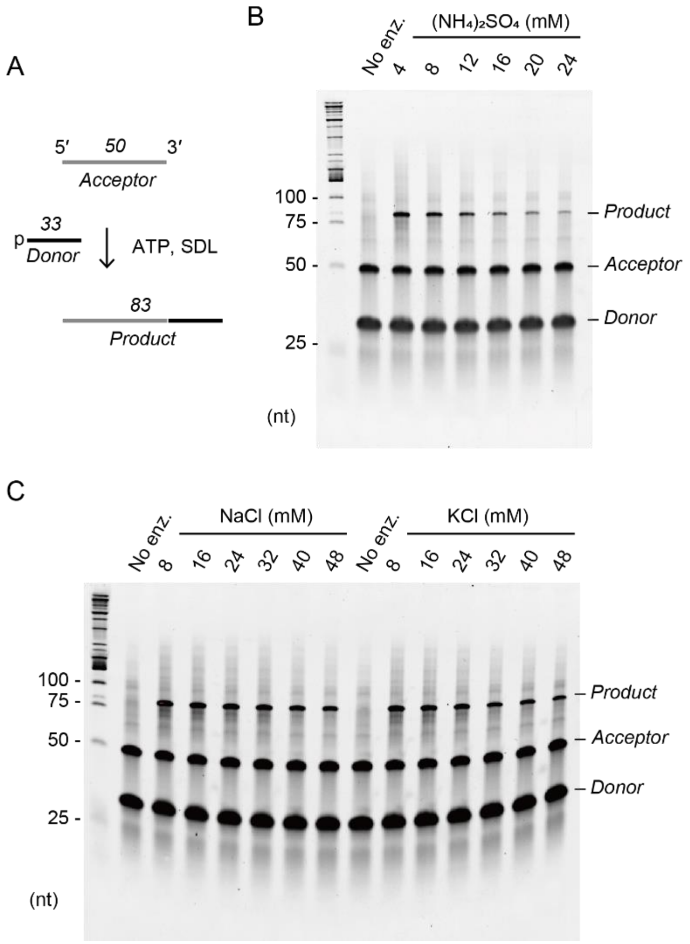

Supplementary Figure S6 The optimum ion concentration for ssDNA ligase. A. The assay design. B and C. Gel image of the denaturing gel electrophoresis of the reaction. Reaction solutions containing indicated ions, 50 mM Tris-HCl, pH8.5, 5 mM $MgCl_2$, 0.05% (v/v) Tritton X-100, 400 μM ATP, 16% (w/v) PEG #6000, 5 pmol acceptor (N50), 50 pmol donor (P-anti-PEA2-thio5-P) and 2.8 μg (63 pmol) dual-affinity purified AYJ73970 in 25 μL were prepared and incubated at 37°C for 1 h. The procedures used to purify the reaction and gel electrophoresis were the same as those in Supplementary Figure S3.

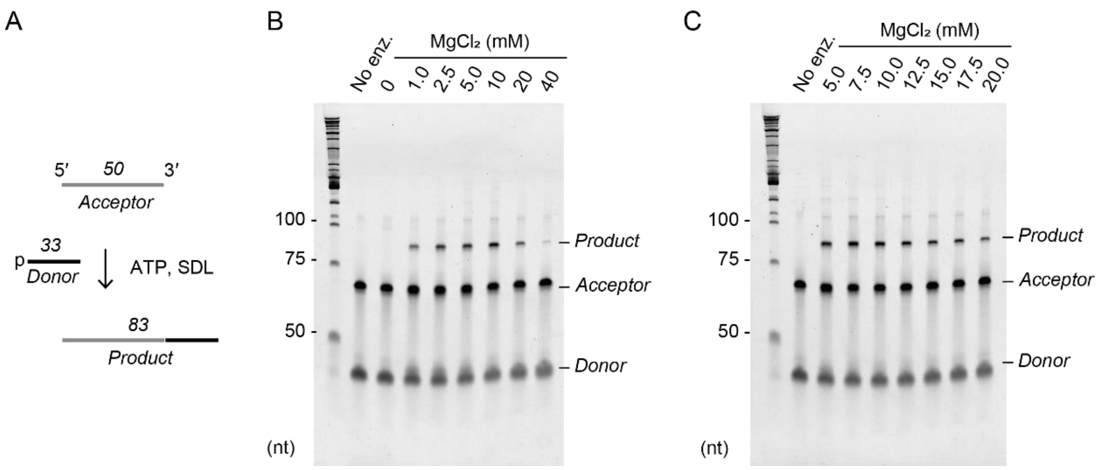

Supplementary Figure S7 The optimum concentration of magnesium chloride for ssDNA ligase. A. The assay design. B and C. Gel image of the denaturing gel electrophoresis of the reaction. Reaction solutions containing 50 mM Tris-HCl, pH8.5, the indicated concentration of $MgCl_2$, 0.05% (v/v) Tritton X-100, 400 μM ATP, 16% (w/v) PEG #6000, 5 pmol acceptor (N50), 50 pmol donor (P-anti-PEA2-thio5-P), and 2.8 μg (63 pmol) dual-affinity purified AYJ73970 in 25 μL were prepared and incubated at 37°C for 1 h. The procedures used to purify the reaction and gel electrophoresis were the same as those in Supplementary Figure S3.

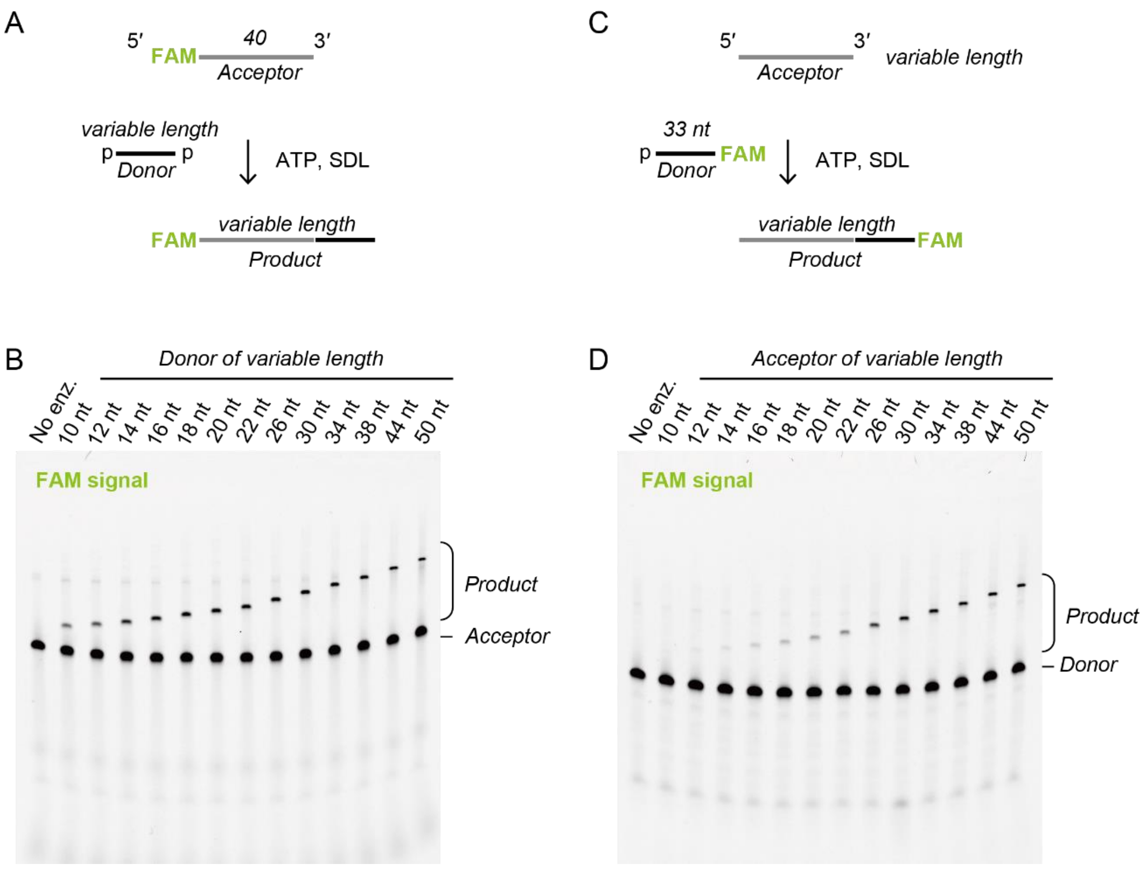

Supplementary Figure S8. Minimum size requirements of donor and acceptor for ssDNA ligase. A and C. The assay design used to investigate the length required for a donor (A) and acceptor (C). B and D. Gel image of the denaturing gel electrophoresis of the reaction. Reaction solutions containing 50 mM Tris-HCl, pH8.5, 10 mM $MgCl_2$, 0.05% (v/v) Tritton X-100, 400 µM ATP, 30% (v/v) PEG #600, oligonucleotides (see below), and 2.8 µg (63 pmol) dual-affinity purified AYJ73970 in 25 µL were prepared and incubated at 37°C for 1 h. The reaction solution was diluted with formamide at a 1:9 ratio, heat incubated at 95°C for 3 min, and loaded on 10% Novex TBE-Urea Gels. After running, the gel was photographed without staining with the ChemiDoc Touch imaging system. To investigate donor length, 50 pmol of an acceptor (FAM-N40, Supplementary Table S1) and 100 pmol of the donor (P-NX-P, X denotes the oligonucleotide length, Supplementary Table S1) were used. For the acceptor length, 50 pmol of the donor (P-anti-PEA2-thio5-FAM, Supplementary Table S1) and 100 pmol of the acceptor (NX, X indicates the length of the oligonucleotides, Supplementary Table S1) were used.

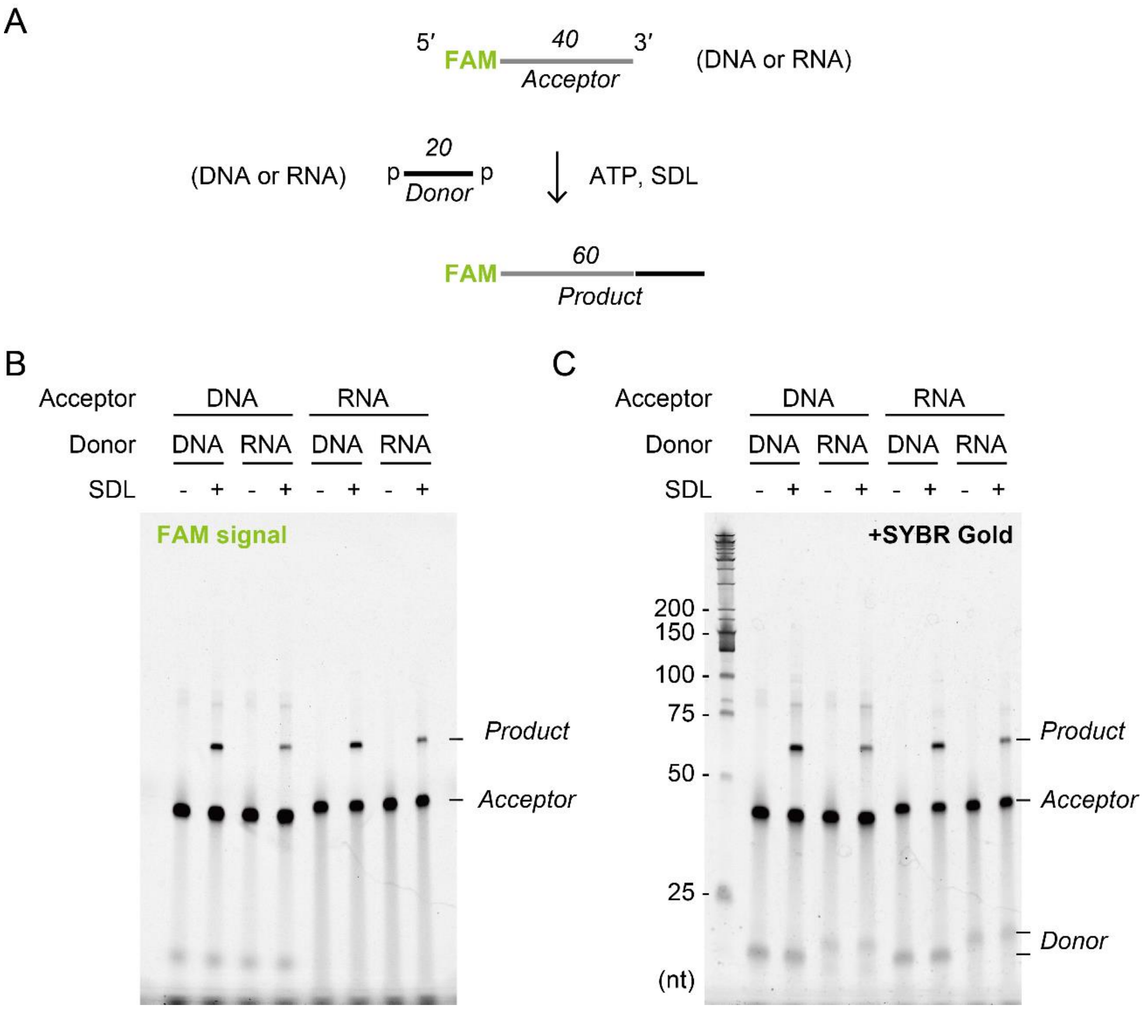

Supplementary Figure S9 The reactivity of ssDNA ligase on RNA. A. The assay design. B and C. Gel image of the denaturing gel electrophoresis of the reaction. Reaction solutions containing 50 mM Tris-HCl, pH8.5, 10 mM $MgCl_2$, 0.05% (v/v) Tritton X-100, 400 μM ATP, 30% (v/v) PEG #600, 100 pmol donor (DNA or RNA, P-N20-P or P-rN20-P, Supplementary Table S1), 100 pmol acceptor (DNA or RNA, FAM-N40 or FAM-rN40, Supplementary Table S1), and 5 μg (114 pmol) dual-affinity purified AYJ73970 in 25 μL were prepared and incubated at 37°C for 1 h. The reaction solution (1 μL) was mixed with 10 μL of formamide and incubated at 70°C for 5 min. Then, each sample was loaded on a 10% Novex TBE-Urea Gel. After running, the gel was photographed without staining with the ChemiDoc Touch imaging system (B). Then, the image after SYBR Gold staining was captured (C).

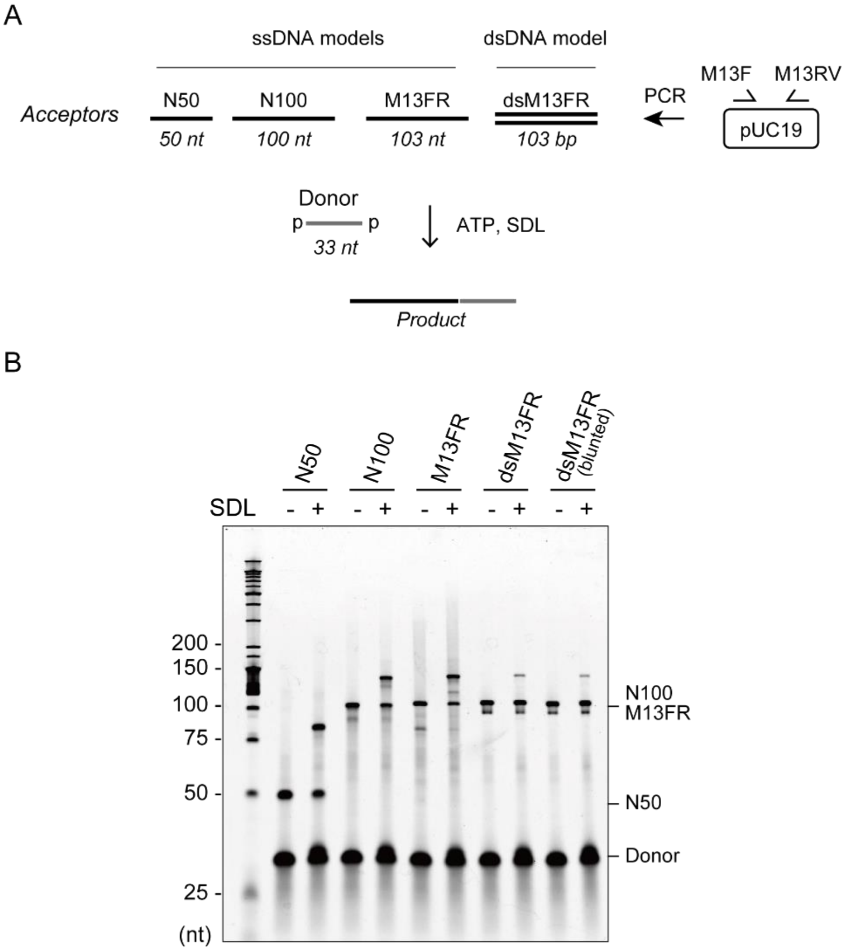

Supplementary Figure S10 Ligation activity of SDL on adaptor tagging of ssDNA and dsDNA A. The assay design. dsDNA was prepared with PCR. Following PCR, the dsDNA was divided into two equal parts. After the PCR, one half was used directly for subsequent processes, and the other half was treated with Klenow to blunt the end. All other ssDNA acceptors are chemically synthesized. B. Gel image of the denaturing gel electrophoresis of the reaction. Reaction solutions containing 50 mM Tris-HCl, pH8.5, 10 mM $MgCl_2$, 0.05% (v/v) Tritton X-100, 400 μM ATP, 30% (v/v) PEG #600, 50 pmol of the donor (P-anti-PEA2-thio5-P), 80 ng of the acceptor (ssDNA or dsDNA), and 2.8 μg (63 pmol) dual-affinity purified AYJ73970 in 25 μL were prepared and incubated at 37°C for 1 h. The reaction (1 μL) solution was mixed with 10 μL of formamide and incubated at 70°C for 5 min. Then, the samples were loaded on a 10% Novex TBE-Urea Gel. After running, the gel was stained with SYBR Gold staining, and the gel image was captured. N50, N100, and M13FR indicate the DNA used (Supplementary Table S1).

For dsM13FR preparations, 50 μL reaction solutions containing 1×ExTaq Buffer (Takara Bio Inc), 200 μM dNTPs, 0.6 μM M13F (Supplementary Table S1), 0.6 μM M13RV (Supplementary Table S1), 10 pg of pUC19 (NEB), and 5 unit of ExTaq HS (Takara Bio Inc.) were prepared and PCR-amplified with 25 cycles of three-step incubations of 15 s: 95°C, 55°C, and 72°C. For blunted one, five units of Klenow fragment (NEB) were added to the reaction, followed by further incubation at 37°C for 10 min. The PCR-amplified DNA was purified using a QIAQuick PCR purification kit (Qiagen). The DNA concentration was determined using a Qubit dsDNA BR assay kit (Thermofisher Scientific).

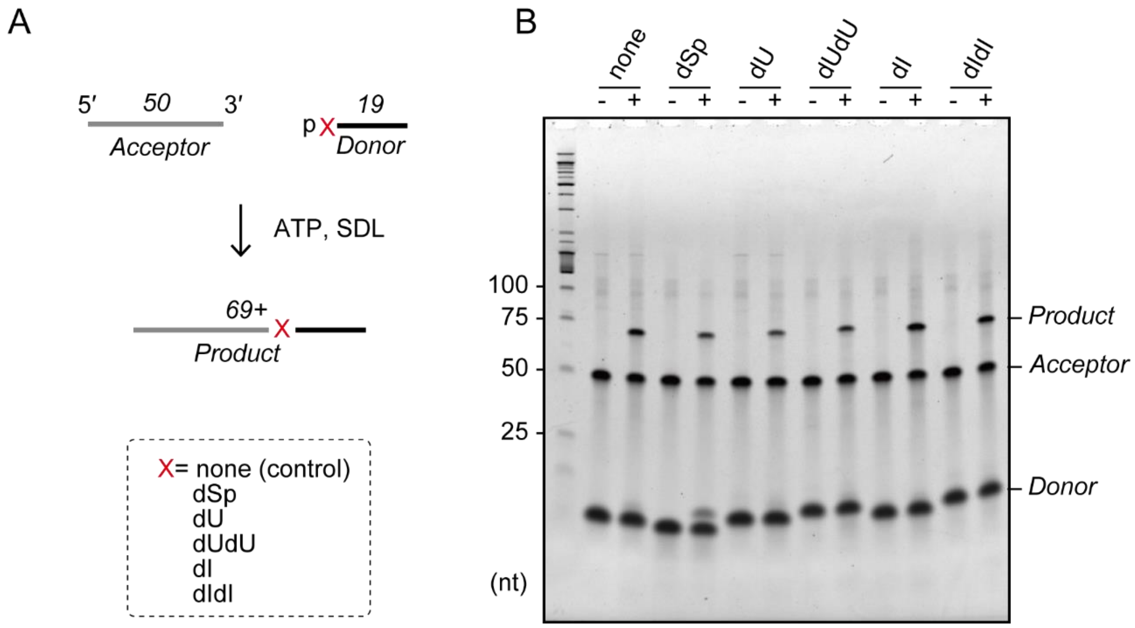

Supplementary Figure S11 The effects of modified bases on the single-strand ligation activity of SDL A. The assay design. B. Gel image of the denaturing gel electrophoresis of the reaction. Reaction solutions containing 50 mM Tris-HCl, pH8.5, 10 mM $MgCl_2$, 0.05% (v/v) Triton X-100, 400 μM ATP, 30% (w/v) PEG #600, 5 pmol acceptor (N50), 25 pmol indicated donor (P-XAR-NH2, X denotes the modified base(s), Supplementary Table S1) and 5 μg (114 pmol) dual-affinity purified AYJ73970 in 25 μL were prepared and incubated at 37°C for 1 h. The procedures used to purify the reaction and gel electrophoresis were the same as those in Supplementary Figure S3. The symbols denote the following: dSp: dSpacer, an abasic site mimic; dU: deoxyuridine; dI: deoxyinosine.

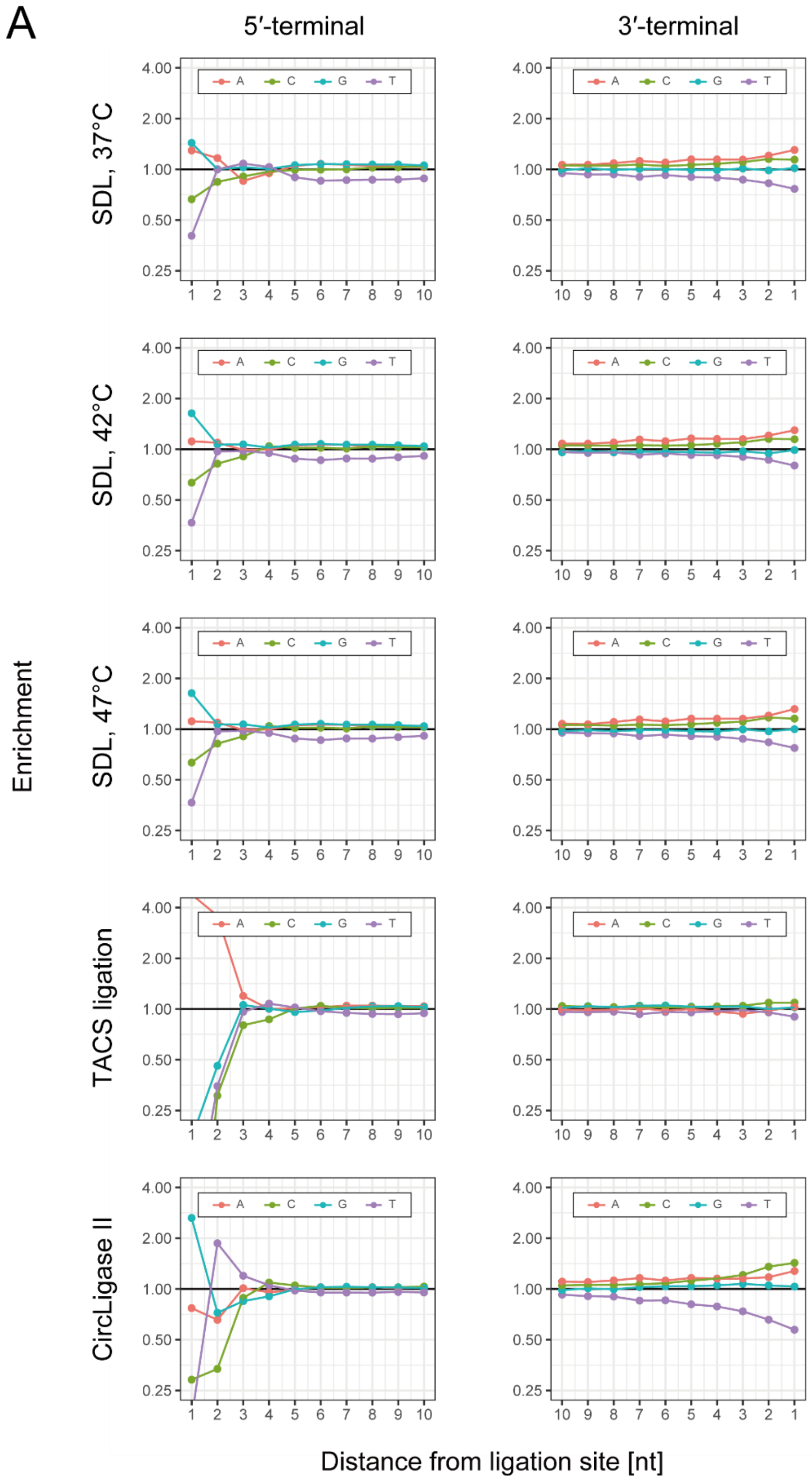

(Continues on next page)

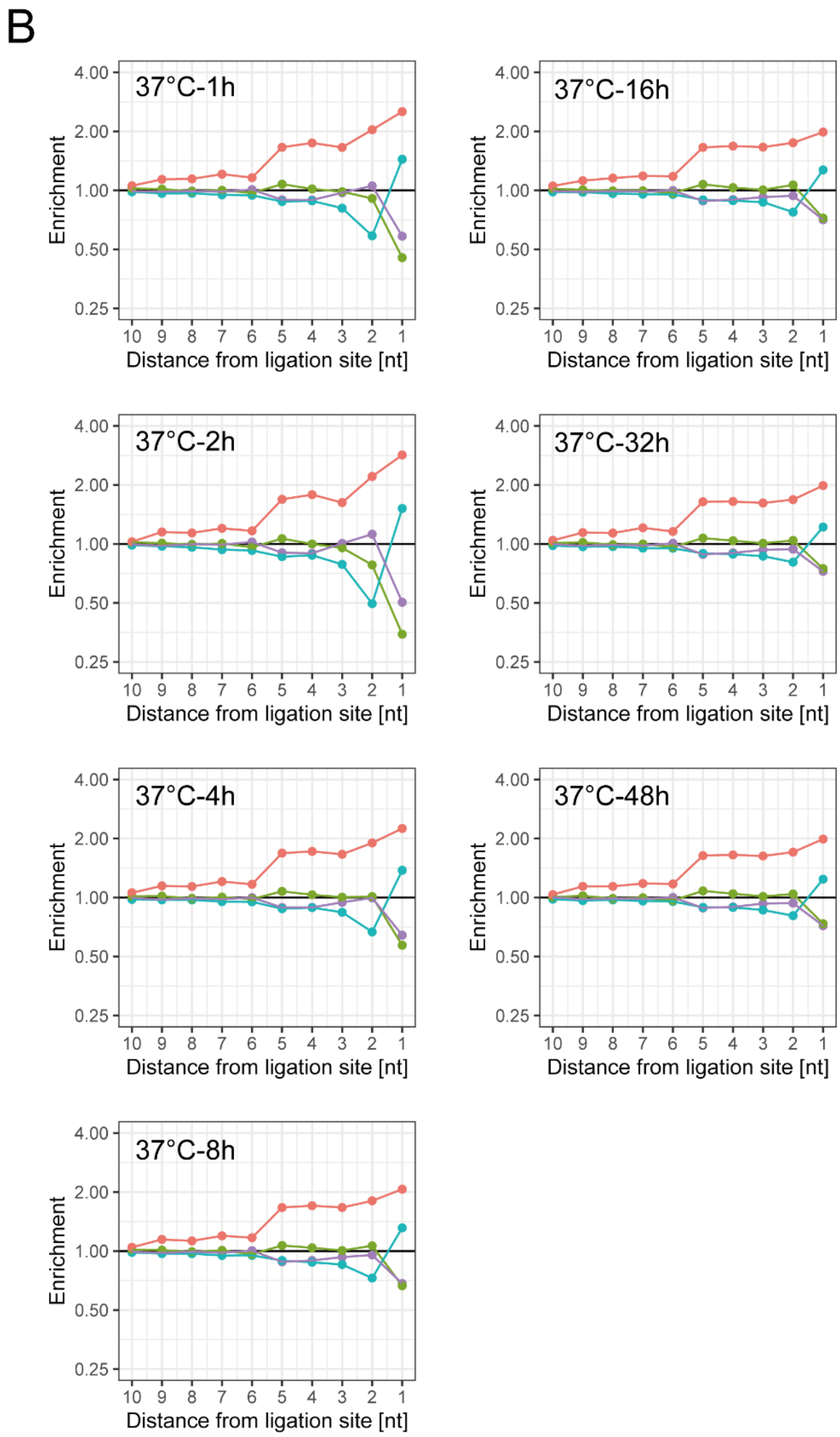

(Continues on next page)

C

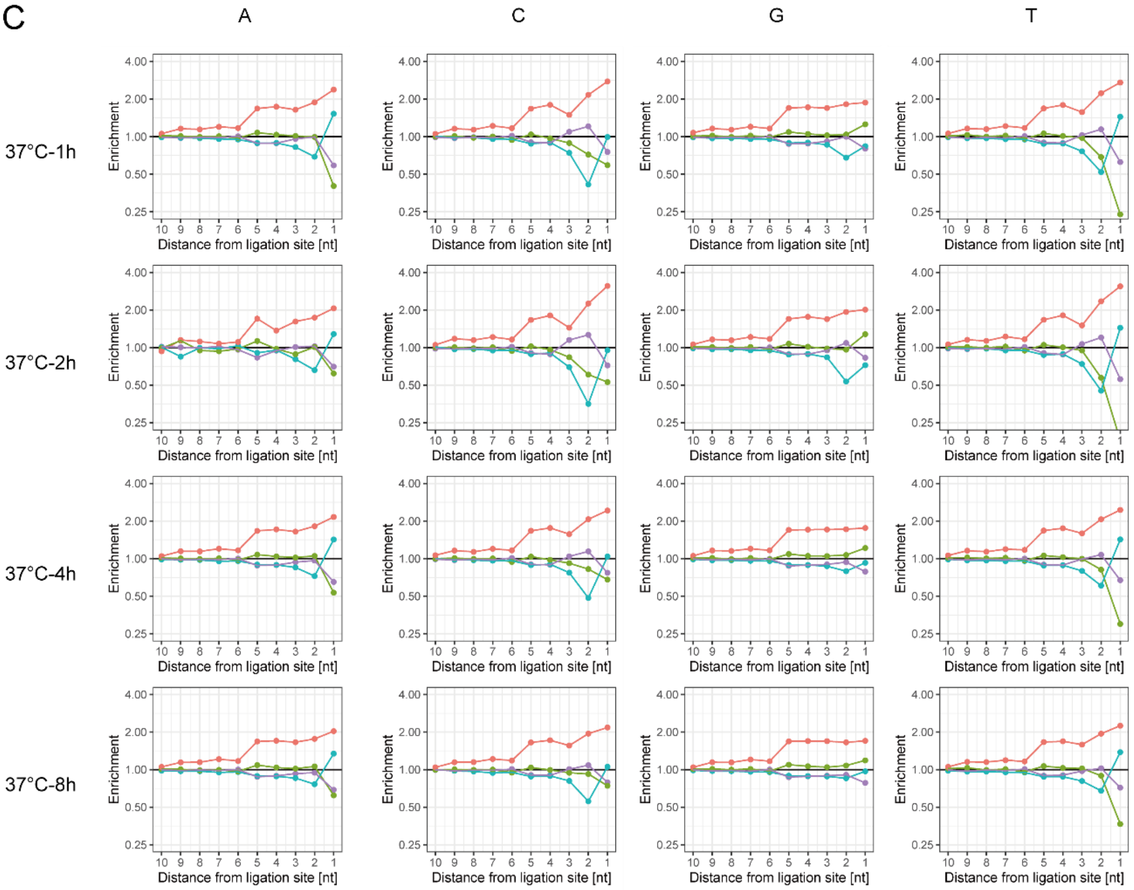

Supplementary Figure S12 Terminal nucleotides-dependent ligation bias A. Sequencing-based analysis of the nucleotide preference of ssDNA ligation techniques. Preferences of 5′- and 3′-nucleobases were determined by sequencing ligation products as described in Figure 3D–3K. The reaction conditions used are described in Supplementary Methods. B. The effect of incubation time on the nucleotide preferences. C. The effect of 5′-terminal nucleobase of adaptor on the nucleotide bias. The nucleotides at the 5′-terminal of the adaptors are indicated at the top of the panels. The durations of incubations are indicated on the left side of the panels.

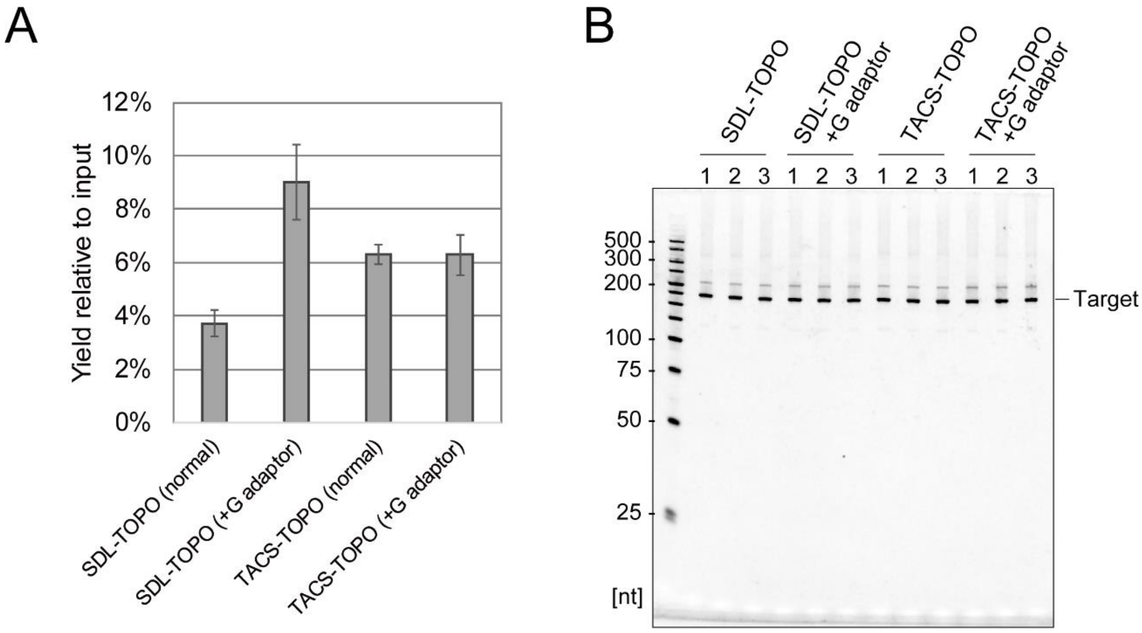

Supplementary Figure S13 Comparison of SDL-TOPO and TACS-TOPO The yields (A) and gel image (B) of denaturing gel electrophoresis of PCR-amplified SDL-TOPO and TACS-TOPO libraries prepared from 1 pmol of model DNA (N50) are shown. The expected sizes of libraries are 176 bp and 178 bp for SDL-TOPO and TACS-TOPO, respectively. For the normal libraries, P-AR-thio5-NH2 was used for the first adaptor tagging with SDL to the acceptor. For +G adaptor libraries, PG-AR-this5-NH2 was used instead (Supplementary Table S1).

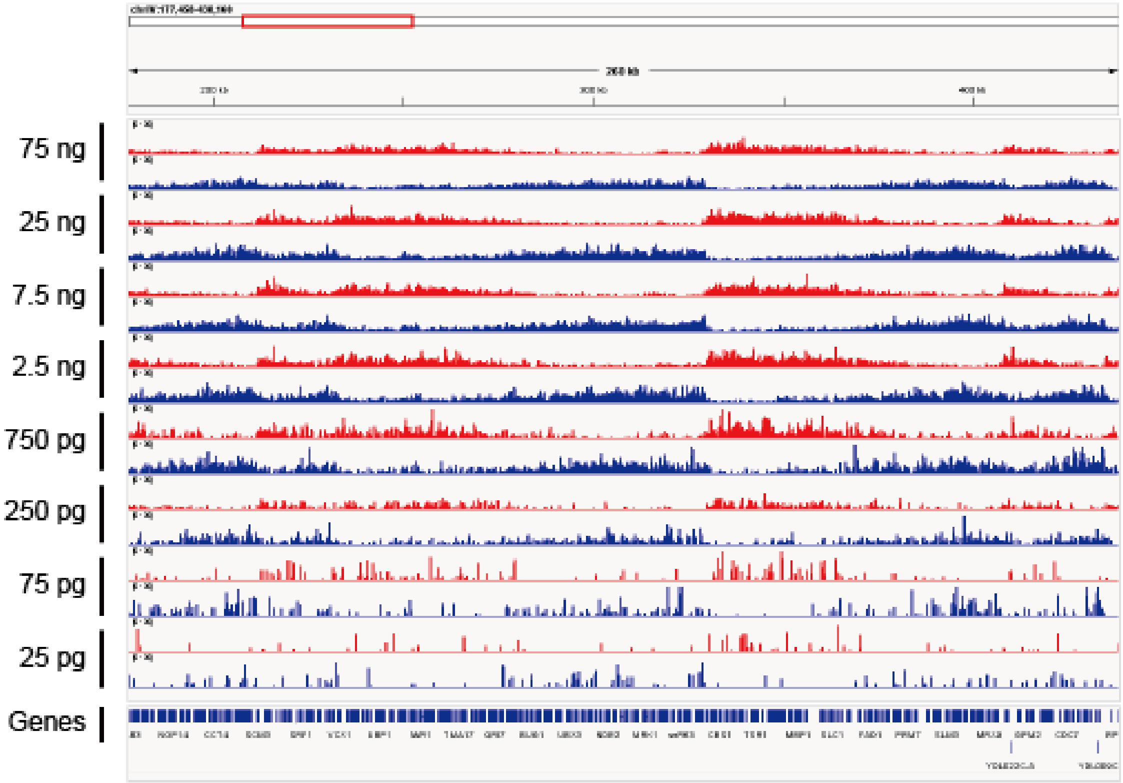

Supplementary Figure S14 A highly sensitive detection of lagging strand of *cdc9* mutant of budding yeast cells The sequencing libraries were prepared from the indicated amount of genomic DNA extracted from the *cdc9* mutant of budding yeast cells. The mapped read depth was visualized with IGV. Read depths on the upper and bottom strands are shown in red and blue, respectively. For details, see Supplementary Methods.

Supplementary Methods

Specific solutions and procedures

The 10×Oligo Hyb comprised 100 mM Tris-HCl, pH 7.5, and 1.6 M NaCl. The 5×TOPO activation buffer comprised 100 mM Tris-Acetate, pH 8.0, 250 mM potassium acetate, and 50 mM magnesium acetate. Buffer 300 comprised 50 mM Tris-HCl, pH 8.0, and 300 mM NaCl. Buffer 400 comprised 50 mM Tris-HCl, pH 8.0, and 400 mM NaCl. Buffer 1000 comprised 50 mM Tris-HCl, pH 8.0, and 1000 mM NaCl. The 10×SDL buffer comprised 500 mM Tris-HCl, pH 8.5, 100 mM $MgCl_2$, and 0.5% (v/v) Triton X-100. Microman E (Gilson) was used to dispense viscous PEG liquids (Nacalai Tesque). The 300-bp cutoff solution comprised 50 mM Tris-HCl, pH 8.0, 1 M NaCl, and 19% (w/v) PEG #400 (Nacalai Tesque). The 400-bp cutoff solution comprised 50 mM Tris-HCl, pH 8.0, 1 M NaCl, and 18% (w/v) PEG #400.

Reaction conditions for panels of Figure 2C

CircLigase II: A 50-μL reaction solution containing 1×CircLigase II reaction buffer (Lucigen), 20% (w/v) PEG #6000, 2.5 mM $MnCl_2$, 6 pmol of N100, 30 pmol pAR-thio5-NH2, 30 units of CircLigase II, and the indicated concentration of ATP was incubated at 60°C. After the indicated periods, 10 μL of the reaction solution was obtained and analyzed as described below.

TS2126 RNA ligase: A 50-μL reaction solution containing 1×TACS buffer (50 mM HEPES-NaOH, pH 7.5, 5 mM $MgCl_2$, and 0.05% Triton-X 100), 20% (w/v) PEG #6000, 6 pmol of N100, 30 pmol pAR-thio5-NH2, 2 μg (46 pmol) TS2126 RNA ligase, and the indicated concentration of ATP was incubated at 65°C. After indicated periods, 10 μL of reaction was obtained and analyzed as described below.

TACS ligation: A 50-μL reaction solution containing 1×TACS buffer, 20%(w/v) PEG #6000, 6 pmol of N100, 30 pmol pAR-thio5-NH2, 2 μg (46 pmol) TS2126 RNA ligase, 45 units of TdT (Takara Bio Inc.) and indicated concentration of ATP was incubated at 37°C for 30 min. Then, the reaction was incubated at 65°C. At the indicated time point, 10 μL of reaction was obtained and analyzed as described below.

SDL: A 50-μL reaction solution containing 1×SDL buffer, 30%(w/v) PEG #600, 6 pmol of N100, 30 pmol pAR-thio5-NH2, 5 μg (114 pmol) SDL, and the indicated concentration of ATP was incubated at 37°C for 30 min. Then, the reaction solution was incubated at

65°C. At the indicated time point, 10 µL of reaction was obtained and analyzed as described below.

Analysis of reaction product: The collected sample was mixed with buffer B2, 20 µL Axy Prep Mag PCR clean, and 100 µL isopropanol. After incubating at ambient temperatures for 5 min, the beads were washed with 70% ethanol. Then, purified DNA was eluted with 10 mM Tris-Acetate, pH 8.0, and analyzed on 10% Novex TBE-Urea Gels with SYBR Gold gel stain.

Library preparation from genomic DNA extracted from budding yeast *cdc9* mutant cells

Strain, culture Genomic DNA extraction: Budding yeast strain BY20149 (*MAT*a *ade2-1 can1-100 his3-11,15 leu2-3,112 trp1-1 ura3-1 cdc9-7*, W303 background) was provided by the National Bio-Resource Project (NBRP) – Yeast, Japan. A single colony was inoculated in 10 mL YPD media (2%(w/v) glucose, 2% (w/v) peptone, and 1% (w/v) yeast extract) and cultivated at 30°C overnight while shaking at 250 RPM. The 1 mL of grown cells were diluted 1:9 with the same media and cultivated again at 30°C for 2 h. Cells were collected with centrifugation, washed once with water, and served for genomic DNA extraction using Genomic-tip 100/G from Qiagen following the yeast-specific protocol provided by the manufacturer.

SDL-TOPO protocol: A 14.5 µL reaction containing the indicated amount of genomic DNA, 2.5 µL 10x SDL buffer, and 1 µL rSAP (NEB) was prepared and incubated at 37°C for 15 min followed by 95°C for 5 min. Then, the reaction was supplemented with 7.5 µL PEG600, 1 µL each 10 mM ATP, 10 µM P-AR-thio5-NH2, and 5 mg/ml SDL. The reaction was incubated at 37°C for 1 h and then 95 °C for 5 min. The following procedures for replication and purification, second adaptor tagging, and indexing were conducted as described in the material and methods section of the main text, except for the beads wash solution used. In this case, we used a solution containing 19% (v/v) PEG #400, 1 M NaCl, and 50 mM Tris-HCl, pH 8.0. Washing the beads with this solution removes DNA fragments shorter than 300 bp.

Sequencing and data analysis: The libraries were sequence determined with the Illumina iSeq 100 system, and obtained reads were mapped on the sacCer3 reference genome with bowtie2 (1). The exported bam file was converted to a bigwig file using bedtools (2) and bedGraphToBigWig. The bigwig file was visualized with IGV (3).

References

1. Langmead B. and Salzberg S.L., Fast gapped-read alignment with Bowtie 2, Nature Methods, 2012, 9, 357-359, 10.1038/nmeth.1923

2. Quinlan A.R. and Hall I.M., BEDTools: a flexible suite of utilities for comparing genomic features, Bioinformatics, 2010, 26, 841-842, 10.1093/bioinformatics/btq033

3. Robinson J.T., Thorvaldsdóttir H., Winckler W., Guttman M., Lander E.S., Getz G., and Mesirov J.P., Integrative genomics viewer, Nature Biotechnology, 2011, 29, 24-26, 10.1038/nbt.1754

Supplementary Sequences

The basic expression vector used in the current study

>pET28a-StrepSL
TGGCGAATGGGACGCGCCCTGTAGCGGCGCATTAAGCGCGGCGGGTGTGGTGGTTACGCG
CAGCGTGACCGCTACACTTGCCAGCGCCCTAGCGCCCGCTCCTTTCGCTTTCTTCCCTTC
CTTTCTCGCCACGTTCGCCGGCTTTCCCCGTCAAGCTCTAAATCGGGGGCTCCCTTTAGG
GTTCCGATTTAGTGCTTTACGGCACCTCGACCCCAAAAAACTTGATTAGGGTGATGGTTC
ACGTAGTGGGCCATCGCCCTGATAGACGGTTTTTCGCCCTTTGACGTTGGAGTCCACGTT
CTTTAATAGTGGACTCTTGTTCCAAACTGGAACAACACTCAACCCTATCTCGGTCTATTC
TTTTGATTTATAAGGGATTTTGCCGATTTCGGCCTATTGGTTAAAAAATGAGCTGATTTA
ACAAAAATTTAACGCGAATTTTAACAAAATATTAACGTTTACAATTTCAGGTGGCACTTT
TCGGGGAAATGTGCGCGGAACCCCTATTTGTTTATTTTTCTAAATACATTCAAATATGTA
TCCGCTCATGAATTAATTCTTAGAAAAACTCATCGAGCATCAAATGAAACTGCAATTTAT
TCATATCAGGATTATCAATACCATATTTTTGAAAAAGCCGTTTCTGTAATGAAGGAGAAA
ACTCACCGAGGCAGTTCCATAGGATGGCAAGATCCTGGTATCGGTCTGCGATTCCGACTC
GTCCAACATCAATACAACCTATTAATTTCCCCTCGTCAAAAATAAGGTTATCAAGTGAGA
AATCACCATGAGTGACGACTGAATCCGGTGAGAATGGCAAAAGTTTATGCATTTCTTTCC
AGACTTGTTCAACAGGCCAGCCATTACGCTCGTCATCAAAATCACTCGCATCAACCAAAC
CGTTATTCATTCGTGATTGCGCCTGAGCGAGACGAAATACGCGATCGCTGTTAAAAGGAC
AATTACAAACAGGAATCGAATGCAACCGGCGCAGGAACACTGCCAGCGCATCAACAATAT
TTTCACCTGAATCAGGATATTCTTCTAATACCTGGAATGCTGTTTTCCCGGGGATCGCAG
TGGTGAGTAACCATGCATCATCAGGAGTACGGATAAAATGCTTGATGGTCGGAAGAGGCA
TAAATTCCGTCAGCCAGTTTAGTCTGACCATCTCATCTGTAACATCATTGGCAACGCTAC
CTTTGCCATGTTTCAGAAACAACTCTGGCGCATCGGGCTTCCCATACAATCGATAGATTG
TCGCACCTGATTGCCCGACATTATCGCGAGCCCATTTATACCCATATAAATCAGCATCCA
TGTTGGAATTTAATCGCGGCCTAGAGCAAGACGTTTCCCGTTGAATATGGCTCATAACAC
CCCTTGTATTACTGTTTATGTAAGCAGACAGTTTTATTGTTCATGACCAAAATCCCTTAA
CGTGAGTTTTCGTTCCACTGAGCGTCAGACCCCGTAGAAAAGATCAAAGGATCTTCTTGA
GATCCTTTTTTTCTGCGCGTAATCTGCTGCTTGCAAACAAAAAAACCACCGCTACCAGCG
GTGGTTTGTTTGCCGGATCAAGAGCTACCAACTCTTTTTCCGAAGGTAACTGGCTTCAGC
AGAGCGCAGATACCAAATACTGTCCTTCTAGTGTAGCCGTAGTTAGGCCACCACTTCAAG
AACTCTGTAGCACCGCCTACATACCTCGCTCTGCTAATCCTGTTACCAGTGGCTGCTGCC
AGTGGCGATAAGTCGTGTCTTACCGGGTTGGACTCAAGACGATAGTTACCGGATAAGGCG
CAGCGGTCGGGCTGAACGGGGGGTTCGTGCACACAGCCCAGCTTGGAGCGAACGACCTAC
ACCGAACTGAGATACCTACAGCGTGAGCTATGAGAAAGCGCCACGCTTCCCGAAGGGAGA
AAGGCGGACAGGTATCCGGTAAGCGGCAGGGTCGGAACAGGAGAGCGCACGAGGGAGCTT
CCAGGGGGAAACGCCTGGTATCTTTATAGTCCTGTCGGGTTTCGCCACCTCTGACTTGAG
CGTCGATTTTTGTGATGCTCGTCAGGGGGGCGGAGCCTATGGAAAAACGCCAGCAACGCG
GCCTTTTTACGGTTCCTGGCCTTTTGCTGGCCTTTTGCTCACATGTTCTTTCCTGCGTTA
TCCCCTGATTCTGTGGATAACCGTATTACCGCCTTTGAGTGAGCTGATACCGCTCGCCGC
AGCCGAACGACCGAGCGCAGCGAGTCAGTGAGCGAGGAAGCGGAAGAGCGCCTGATGCGG
TATTTTCTCCTTACGCATCTGTGCGGTATTTCACACCGCATATATGGTGCACTCTCAGTA
CAATCTGCTCTGATGCCGCATAGTTAAGCCAGTATACACTCCGCTATCGCTACGTGACTG
GGTCATGGCTGCGCCCCGACACCCGCCAACACCCGCTGACGCGCCCTGACGGGCTTGTCT

GCTCCCGGCATCCGCTTACAGACAAGCTGTGACCGTCTCCGGGAGCTGCATGTGTCAGAG
GTTTTCACCGTCATCACCGAAACGCGCGAGGCAGCTGCGGTAAAGCTCATCAGCGTGGTC
GTGAAGCGATTCACAGATGTCTGCCTGTTCATCCGCGTCCAGCTCGTTGAGTTTCTCCAG
AAGCGTTAATGTCTGGCTTCTGATAAAGCGGGCCATGTTAAGGGCGGTTTTTTCCTGTTT
GGTCACTGATGCCTCCGTGTAAGGGGGATTTCTGTTCATGGGGGTAATGATACCGATGAA
ACGAGAGAGGATGCTCACGATACGGGTTACTGATGATGAACATGCCCGGTTACTGGAACG
TTGTGAGGGTAAACAACTGGCGGTATGGATGCGGCGGGACCAGAGAAAAATCACTCAGGG
TCAATGCCAGCGCTTCGTTAATACAGATGTAGGTGTTCCACAGGGTAGCCAGCAGCATCC
TGCGATGCAGATCCGGAACATAATGGTGCAGGGCGCTGACTTCCGCGTTTCCAGACTTTA
CGAAACACGGAAACCGAAGACCATTCATGTTGTTGCTCAGGTCGCAGACGTTTTGCAGCA
GCAGTCGCTTCACGTTCGCTCGCGTATCGGTGATTCATTCTGCTAACCAGTAAGGCAACC
CCGCCAGCCTAGCCGGGTCCTCAACGACAGGAGCACGATCATGCGCACCCGTGGGGCCGC
CATGCCGGCGATAATGGCCTGCTTCTCGCCGAAACGTTTGGTGGCGGGACCAGTGACGAA
GGCTTGAGCGAGGGCGTGCAAGATTCCGAATACCGCAAGCGACAGGCCGATCATCGTCGC
GCTCCAGCGAAAGCGGTCCTCGCCGAAAATGACCCAGAGCGCTGCCGGCACCTGTCCTAC
GAGTTGCATGATAAAGAAGACAGTCATAAGTGCGGCGACGATAGTCATGCCCCGCGCCCA
CCGGAAGGAGCTGACTGGGTTGAAGGCTCTCAAGGGCATCGGTCGAGATCCCGGTGCCTA
ATGAGTGAGCTAACTTACATTAATTGCGTTGCGCTCACTGCCCGCTTTCCAGTCGGGAAA
CCTGTCGTGCCAGCTGCATTAATGAATCGGCCAACGCGCGGGGAGAGGCGGTTTGCGTAT
TGGGCGCCAGGGTGGTTTTTCTTTTCACCAGTGAGACGGGCAACAGCTGATTGCCCTTCA
CCGCCTGGCCCTGAGAGAGTTGCAGCAAGCGGTCCACGCTGGTTTGCCCCAGCAGGCGAA
AATCCTGTTTGATGGTGGTTAACGGCGGGATATAACATGAGCTGTCTTCGGTATCGTCGT
ATCCCACTACCGAGATATCCGCACCAACGCGCAGCCCGGACTCGGTAATGGCGCGCATTG
CGCCCAGCGCCATCTGATCGTTGGCAACCAGCATCGCAGTGGGAACGATGCCCTCATTCA
GCATTTGCATGGTTTGTTGAAAACCGGACATGGCACTCCAGTCGCCTTCCCGTTCCGCTA
TCGGCTGAATTTGATTGCGAGTGAGATATTTATGCCAGCCAGCCAGACGCAGACGCGCCG
AGACAGAACTTAATGGGCCCGCTAACAGCGCGATTTGCTGGTGACCCAATGCGACCAGAT
GCTCCACGCCCAGTCGCGTACCGTCTTCATGGGAGAAAATAATACTGTTGATGGGTGTCT
GGTCAGAGACATCAAGAAATAACGCCGGAACATTAGTGCAGGCAGCTTCCACAGCAATGG
CATCCTGGTCATCCAGCGGATAGTTAATGATCAGCCCACTGACGCGTTGCGCGAGAAGAT
TGTGCACCGCCGCTTTACAGGCTTCGACGCCGCTTCGTTCTACCATCGACACCACCACGC
TGGCACCCAGTTGATCGGCGCGAGATTTAATCGCCGCGACAATTTGCGACGGCGCGTGCA
GGGCCAGACTGGAGGTGGCAACGCCAATCAGCAACGACTGTTTGCCCGCCAGTTGTTGTG
CCACGCGGTTGGGAATGTAATTCAGCTCCGCCATCGCCGCTTCCACTTTTTCCCGCGTTT
TCGCAGAAACGTGGCTGGCCTGGTTCACCACGCGGGAAACGGTCTGATAAGAGACACCGG
CATACTCTGCGACATCGTATAACGTTACTGGTTTCACATTCACCACCCTGAATTGACTCT
CTTCCGGGCGCTATCATGCCATACCGCGAAAGGTTTTGCGCCATTCGATGGTGTCCGGGA
TCTCGACGCTCTCCCTTATGCGACTCCTGCATTAGGAAGCAGCCCAGTAGTAGGTTGAGG
CCGTTGAGCACCGCCGCCGCAAGGAATGGTGCATGCAAGGAGATGGCGCCCAACAGTCCC
CCGGCCACGGGGCCTGCCACCATACCCACGCCGAAACAAGCGCTCATGAGCCCGAAGTGG
CGAGCCCGATCTTCCCCATCGGTGATGTCGGCGATATAGGCGCCAGCAACCGCACCTGTG
GCGCCGGTGATGCCGGCCACGATGCGTCCGGCGTAGAGGATCGAGATCTCGATCCCGCGA
AATTAATACGACTCACTATAGGGGAATTGTGAGCGGATAACAATTCCCCTCTAGAAATAA
TTTTGTTTAACTTTAAGAAGGAGATATACCATGGGCAGCAGCCATCATCATCATCATCAC
AGCAGCGGCCTGGTGCCGCGCGGCAGCCATATGGCTAGCATGACTGGTGGACAGCAAATG

GGTCGCGGATCCGAATTCTGGAGCCATCCGCAGTTTGAAAAATAACAAAGCCCGAAAGGA
AGCTGAGTTGGCTGCTGCCACCGCTGAGCAATAACTAGCATAACCCCTTGGGGCCTCTAA
ACGGGTCTTGAGGGGTTTTTTGCTGAAAGGAGGAACTATATCCGGAT

Sequences synthesized in the current study

>AYJ73970_
CAATCCGCCCTCACTACAACCGATGCTGGGGAAGCCTCCGTTGATCGAGAAAGCATATCG
ACTGGCGAAAGAAACCAAGGCCTTTCTTATTAAACGCAATGGCAACCTGGTGATGTTTAC
GTATGCTTACGTCCATCCCGTAATGGCAGACCCGGAAGCTCGTGAACTTCGGGGCATCAT
TTACGATAGTGTGAGTGGCAACGTTGTATCGCGCCCCTTTCATAAGTTTTTCAACTATCG
GGAACCGCCGTTCGGTCTCACCAGGGAGGGTTTTCCGGACGGGGAAGTTTATCTAGCCCC
GAAAATGGATGGTTACCTCCTTCAGGTGTCATTACTCGAAGACGGTTCCCTTCTGAAAGT
CAGTAGGCGCTCACTGTCGCCGACTCTGCTCAACTCAGTGTTGGAAGAGGTGTGGGGTGA
ACGCGAAGAGGAGGCAACTCGGGAGGTGCTGGAGGGCCTTCCTCGCCCAGCCACACTGCT
GTTCGAAGTAACATCTTCCAAACGACCAGTGCTGGTTCGCCATCAAGCTTCTGAAGTTCG
CTTCCTTGTTGCCAGAGAAATAGGGACCGGCCGTTATCTGCTGCCCGATGAAGTAGGCTG
GCCGATTGAGGGCCAGTCACTGCCCTGGACCCGAGTAAGGATAGATCCGGATAATTTCCT
CGAAGAAATTAAGGATCTGGAAGGAGTAGAGGGTTACGTTGCCTTTTTGCCAGAAAAAAA
TGAATTCGTCAAATTTAAAACAAAATGGGCCTTTAGATTGTCCGGGTTTCTGTTAGACCC
AGTCGAAGGCTTTGTGTCTGCCTACGTTGAAGACCGTGTCGATGATCTTCTGGCAAGCCT
GACAGAACGGCCAGATCTGCAGGAAGCCATACTGAGAGCAAAAGAACTTCTTGATGGGCT
GTTTAGCGAAGCTACCAGCTTAGGAGAGGAGTTACGTGAACGCGGTATTGTACGTAAGGA
GGCGTGGGAACAGGTCAGCAGCTGGGCTAAAGGTAAAGGTTTATCTGGGATTGAAGGGGT
GTTGAAAGAAGTAGCCATGGTCGCATATAGCGGAGGGCAAACGCGGGAAGCCTTTTTAAA
AGGCATTGGTAAAGGTAAATCAGTGGATTTCCTGAAAACATTAGATCTGTTCCCACGCGT
AGGAGAAGAAGTTGGGGCACTACTCTGGCGTCGATGAGGGA

>RTI02525_
CAATCCGCCCTCACTACAACCGATGCGTAAACCGCCGCCGCCGCTGAAAAGAGCGCTGGA
AATAGCCGCACATCCACCATTTCGCGTGCAAAGAGAGGAGGGACTTGTGCTTATTTCTTA
CCGCGAAAAGGGTGGGTTTTCGGAAGCTTCCTGGGATGCCGAGGCCCGCGAATTGCGCGG
TATAGTGTACGCGGAAGCAACGGGAGAGGCAGTGTCCAGACCTTTTCATAGATTTTTTAA
TCTGGGCGACCCGCGCTGTGGTTTCCCGCCGGATCGCCCTCTGAAGACCAATGACTTGCT
AGCGCGCAAAGTTGATGGCTTCCTGTTACAGGTATTTGCTTTCCAGGAAAAACTGTGGTT
TGCATCTCGGGCGGCGCTGCGCTTGAGCAAGGCCGAACATGCTTCCTGGCATCAAGCCTG
GACCCCCCGGCACGACGCGTTTGTGCGACAAGCATTAGAGTCTCTGGGTAGCATTACTCT
TTTGTTTGAAGTGGTTGACCCGAAACGTCTGAGCCTAGAACGACACGAAGAGGCGGCTGC
CGTGCTGTTGGCTATACGCCACATCCCTACTGGACGGTACTGGTTCCCTGGCGCAGCCCC
AGAACTTGACGCTCTGCTGCAGAGTTTCCAGGTGCCTCACGTGAGCTGGCGCCCAGCGGG
CGCGGGTACCTTAGAAGAATTACACCGTCGAGTTCGTAATGAAAAAGATAAGGAAGGTTA
TGTTTTGTGGCTGGAGGAGGGCGATTTTCTGAAGGTTAAAACAGACTGGGCACTGGGTTT
TTCTCGTAAACAGCGGCGGTTTCAAGATCACCTCCGTGAATTTTCGCGCTCCTTAATGGA
GGATCGTTTTGATGATTTTATTGCCGGTCTCTCATCCGAGCCGGAGATGCAGGGCGATTT
TCTGCGTCTTTACCGCCGAATCCGGAACTTGATAGGTGAGACCCTTGCGATGGCAGAGGA

AGTTCGAGGCTTGCCTCGAAAAGAAGCGGTCGCGCTGCTTGAGGATCGCTTGCGCTCTTT
CCCCTTGACTCAAGCCCGCCTATCTCTGGCCCTGGCCGCCTATGAAGGCGGGGAAGAACG
TGCCTGGGCTCGCTTTAAGACTCTGCTGAAAGGTCGTGGCCCGCAGATTCTGGATGCGCT
TTTAGAAGAAGCCCTACTCTGGCGTCGATGAGGGA

>WP_081892735_
CAATCCGCCCTCACTACAACCGATGAGGAAGCCGCCGCCTCCTCTCAAGCGGGCTCTGGA
GATCGCAGCCCATCCGCCTTTCCGCGTTCAGCGCGAGGAGGGCTTGGTCCTGATCAGTTA
TCGCGAAAAAGGAGGGTTCTCCGAGGCCAGTTGGGACGCCGAAGCACGTGAATTACGCGG
AATTGTTTATGCGGAAGCGACTGGTGAGGCTGTTTCCCGTCCGTTCCATCGCTTTTTTAA
TTTGGGCGACCCACGGTGCGGGTTTCCTCCAGACCGCCCGCTTAAAACGAACGACCTACT
AGCTAGAAAAGTGGACGGGTTCTTACTTCAGGTTTTCGCTTTCCAGGAAAAATTATGGTT
TGCATCAAGAGCTGCCCTGCGACTGTCAAAAGCGGAACACGCTAGCTGGCGCCAGGCCTG
GACCCCGAGACACGACGCGTTTGTGCGTCAGGCACTCGAATCACTTGGGAGCATCACCTT
ACTCTTTGAAGTAGTGGACCCAAAGCGTTTATCATTAGAAAGACACGAGGAGGCAGCCGC
GGTCCTGCTCGCCATACGTCATATTCCTACGGGGCGTTATTGGTTCCCAGGTGCGGCCCC
GGAACTCGATGCCCTGCTGCAGTCTTTTCAGGTGCCTCACGTTTCGTGGAGACCAGCGGG
AGCAGGAACACTAGAGGAGTTGCATCGTCGGGTAAGAAATGAAAAGGATAAAGAGGGCTA
TGTTCTGTGGTTAGAAGAAGGGGATTTTCTGAAGGTCAAGACGGACTGGGCACTCGGCTT
CTCACGCAAACAGCGGCGGTTCCAAGACCATCTCCGTGAATTTTCGCGAAGTCTAATGGA
GGATCGCTTTGACGATTTCATTGCAGGTTTATCTTCGGAGCCGGAAATGCAGGGAGATTT
CCTTAGACTGTATAGACGTATTCGCAACCTGATCGGCGAAACACTCGCGATGGCCGAGGA
AGTGCGGGGTTTACCGCGCAAGGAAGCGGTGGCGCTGTTAGAGGGACGTTTACGTTCACT
GCCACTAGCTCAAGCACGCTTGAGCCTGGCGCTTGCGGCTTATGAGGGTGGTGAAGAAAG
AGCATGGGCCCGCTTCAAGACCTTGTTAAAGGGTCGTGGCCCTCAGATTCTGGATGCTCT
CCTAGAAGAAGCACTACTCTGGCGTCGATGAGGGA

>WP_110532308_
CAATCCGCCCTCACTACAACCGATGGAGCGGCCTCCGCCCTTCCTGCCTAAGGCTTTGGC
CCTGGCAGAAAATCCCCCCTTTTTTACACGTCGCAAAGACGGCTTAATTCTGATAGGATA
TCAAGAAGACGGTCTGGTTAGGGACCTGGCGTGGGATGAAGAAACCAGAGAGCTGCGTGG
AATTATTTACCGGGAAGCAACTGGTGCAGTGATATCTCGCCCTTTTCATCGCTTTTTTAA
CGTAGGGGACCCTCGCTCGGGAGTAGATCCGCAGCGTCCTCTGGGGCCGGGCGATCTTCT
GGCGCCTAAAGTGGATGGGCGCCTCTTTCAAGTGTTTTTTTTAGAAGGGAGCCTGCGGCT
CGCGACCCGCGGCTCGCTCGAACTGGCAAAAGCCGAACGTGCTTCATGGCGTAAAGCGTG
GACAGAAGATCATGAACGCCTCGCTCAGCGTGCCCAGGAAGCCCTGGGTCCTGTCACACT
CCTCTTTGAAGTCGTGGATCCTGAACGCCCTATTTTAGAACATCCTCAGGAAGCCGCGGC
AGTTCTGCTGGCAGTCCGTCATGTGGCGACTGGACGATATGGACTTCCCGGTGCCAGTGA
GGTTCTGGAGGAACTCCTGAAAGGTCTGAAAGTTCCACGTATTGCGTGGGCTCCGGCGCC
TCTTGGGGAAACAGTCGGCTCATTACACGAACACATCCGCAAAGAGGCCGGACGCGAGGG
GTTTGTTTTGTGGTTAGCCGAGGGCGATTTTCTGAAGTTAAAGACGGATTGGGCCCTGGG
TATCCCAAAGGAACGGGGTCGCGCTCGCGAATTGCGGCAGGCGTTTCTGGAAGCATTTCT
GGAACGGCGACTGGATGATCTGCTGAGCGCCCTGGAAGACGCACAGGAGAAAGGAGCGAT
GGCGGAGCTGGCCGCAAGGCTAGAAGAGTTGTTAGCAGAAGCCGTTCAGCGCGCAGAGGC
GCATAAAGGCCTTCCGCCAAAGGAGGTATATGTTAGACTGGAGGCCACCCTCCTGGGACA

GCCAATGGGTCCGCTTCGCTTGAGACTTACAATGGGCGCTCATCGCGCCGGCCGTGAAGG
CGCCTGGCGCGTACTTGAGGGCGCGCTGCGCGCTCGGCGTGTCTCCCTTCTGGAAGAACT
CTTAGCATCTCCACCGGGCTTTATGCTGGAACTACTCTGGCGTCGATGAGGGA

>WP_119360902_
CAATCCGCCCTCACTACAACCGATGCTGCTGGGTCCGCCCCCGCCACTGGATCTTGCGCT
GGAAAAAGCACAAACGCCGCCGTTTCTAGTACGAAGGGACGGAGGGTTAGTACTGGTCTC
ATACCGCGAACGAGGTGCAGGTACCCAACCTTCATGGGATCCGGTCACCCGTGAGCTGAG
AGGTATTATTTATCGTGAGGCCACGGGAGAAGTCGTGTCAAGACCGTTTCATCGCTTTTT
CAATTTAGGAGACCCCAGGTGTGGTGTAGACCCTTCAAGGTCCCTGGGTCCGGGTGATTA
CATCGGCCCTAAGGAGGATGGTAGATTACTTCAGGCGTTTTTTCTGGAGGGCGCATTACG
TTTTGCCACGCGTGGGAGCCTTAGACTGTCGCCAGAGGATGAACGGGCGCTGCGCCGGTC
TTGGACATTGGATCACGAAGCACTGGCCGTAAGAGCCCGCGAGGTTCTGGGCCCTGTCAC
ACTACTTTTCGAGGTGCTGGATCCAGAAAGACCGATTATGGTCCGTCCAGAAGCCCCGAA
AGCTGTATTGCTCGCAGTCAGACATATCCCGACCGGTCGATATTGGCTCCCAGGCGTCAG
TCAGGAACTTCGTGAAGTCCTTGGCGGACTTAGGGTGCCTCATGTCGTGTGGGAGCCCGC
AGGTTCAGCTTCTCTGCAGGAAGTTCACCAAAGTATACTGGAACGCGAGGGTGTGGAAGG
CTTCGTAGTGTGGCTAGCACAGGGTGATTTTGTTAAATTGAAGACACGTTGGGCTTTGGG
ATTTACCGGTGAACGTGGCGATCTCGAGCGATTTCTTGAGGCATTTCGAATCGCCTTTTA
CGAAGAACGGCTGGACGACCTGCAGGCTGGCATTGGAGAAGGTCCGTACAGACAAGTGTT
TAGCTTTTTAATGTCACGCCTAGAAGATCTCGTCAGGGAAGCCGTGGCCCAGGCCGAGGC
CGTCAGAGGCCTGCCGCGCAGAGAAGCCTATGAAGGTTTACGACGCGTCCTGGGGGCAAA
ACCTCAGGGTCTCCTGTTATTAAATGTTGCCCTACGGGCGTACGATAGAGGAGAAGGCGA
AGCTTGGGAAGCCTTAAAACGTGAAATGAAGCGGGCCGGACGTGGCATTCTTAAAGATCT
GTTAGCGGAATCGGGGTTTAATGTCGGGGGCATCCCTGTGCGCGGTTGGAGCGCATTTTT
GGAAGAACTACTCTGGCGTCGATGAGGGA

>WP_126206484_
CAATCCGCCCTCACTACAACCGATGTGGGGTAGTCCACCGCCTCTCAAACGGGCGCTGGA
ACTGGCTGCGCGCCCGCCGTTTCAGGTTAGACGCGAAGGTGGGCTGGTGTTAATTTCATA
TCAAGAAAAGGGTGGGTTCTCAGAAATTCCGTGGGACCGTGAAGCGCGTGAACTACGTGG
CATTGTTTACGCAGAAGATACAGGTGAGGCACTGAGCCGTCCGTTCCACCGTTTTTTTAA
TCTTGGAGATCCACGTTGTGCTTTCCCACCGGGTCGCCCTCTGGCTGCCGGCGATCTGCT
CGCCCCTAAAGTTGACGGACGCCTCTTGCAGGTGTTCGCATACCGTGGCACGCCATGGTT
TGCATCACGTGGCAGCCTGCGTCTGTCGGAGGTGGAGGAAGATAGCCGTAGAAAAGCATG
GACCCCGGATCATGATGTATTAGTTCAACGTGCGTTTGCAGCGCTAGGTCCGGTTACGTT
ACTGTTTGAAGTGGTGGACCCAGAACGTCCCGTGGTCGAGCGCCCAAGAGAAGCGGCAGT
AGTACTGCTTGCGGTCCGTCACATACCAACGGGCCGGTATTGGTTTCCGGGTGCCTCTCC
AGAATTGGAAGCACTGCTGGCCGACTTGGATCTGCCGAGGCTGTCTTGGCGCCCAGCTGG
GGAAGGCACCCTTGGTGAATTGCATCAACGTGTGCGGCCTGAGGGTGAGCGTGAAGGGTA
TGTGCTGTGGTTAGCGGAAGGGGACTTTGTCAAACTGAAGACAGACTGGGCCCTGGGCTT
CACTCGTAAACGACAGTCCTTTCGGAAACGCTTACAGGAATTTGCACAAGCATTGCTCGA
AGATCGCCTAGACGATCTGCTGGCTGGTGGTGCTGATGAACCGGAAAGTCAGCGTATCTT
CTTGCGCTTATATCGAAGGCTGCAGGGCTTAGTAGCGCAGGCCACGGCGTTGGCAGGCGA
AGCCCGTGACCTATCTCGTAAGGAAGCATATGCACTTTTCGAAGAACGTTTAGGGTCTAG

CCGATTTCTCCTGGATTTAGCACTACGCGCGCACGAGGGTGGCGAAGATCGTGCGTGGTC
GCGTCTAAAGGATGTACTGCGCAAAAAGGGTCCCGAATTCCTGGAGGTCCTTTGGGAAGA
GGCTCTACTCTGGCGTCGATGAGGGA

Original gel images

Figure 1C

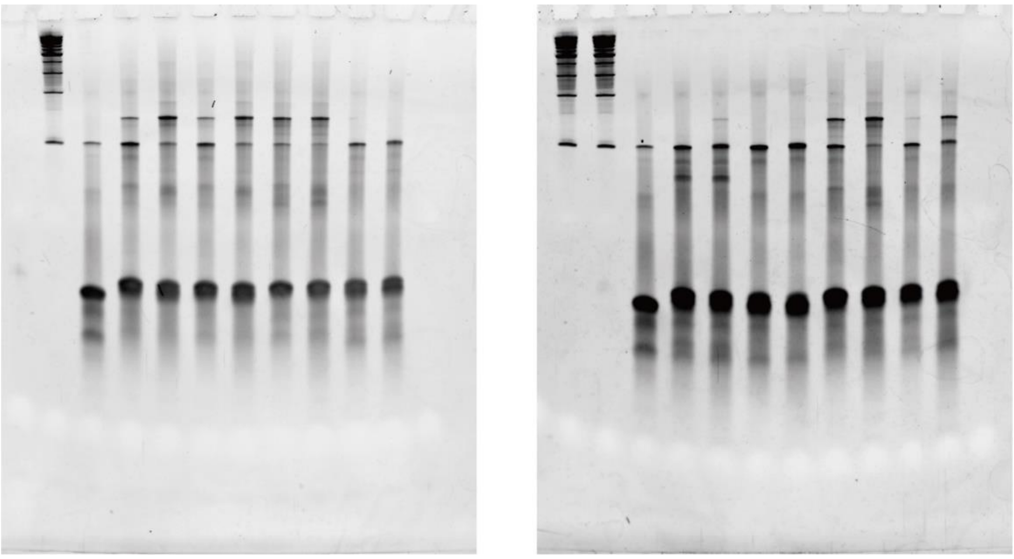

Figure 2C

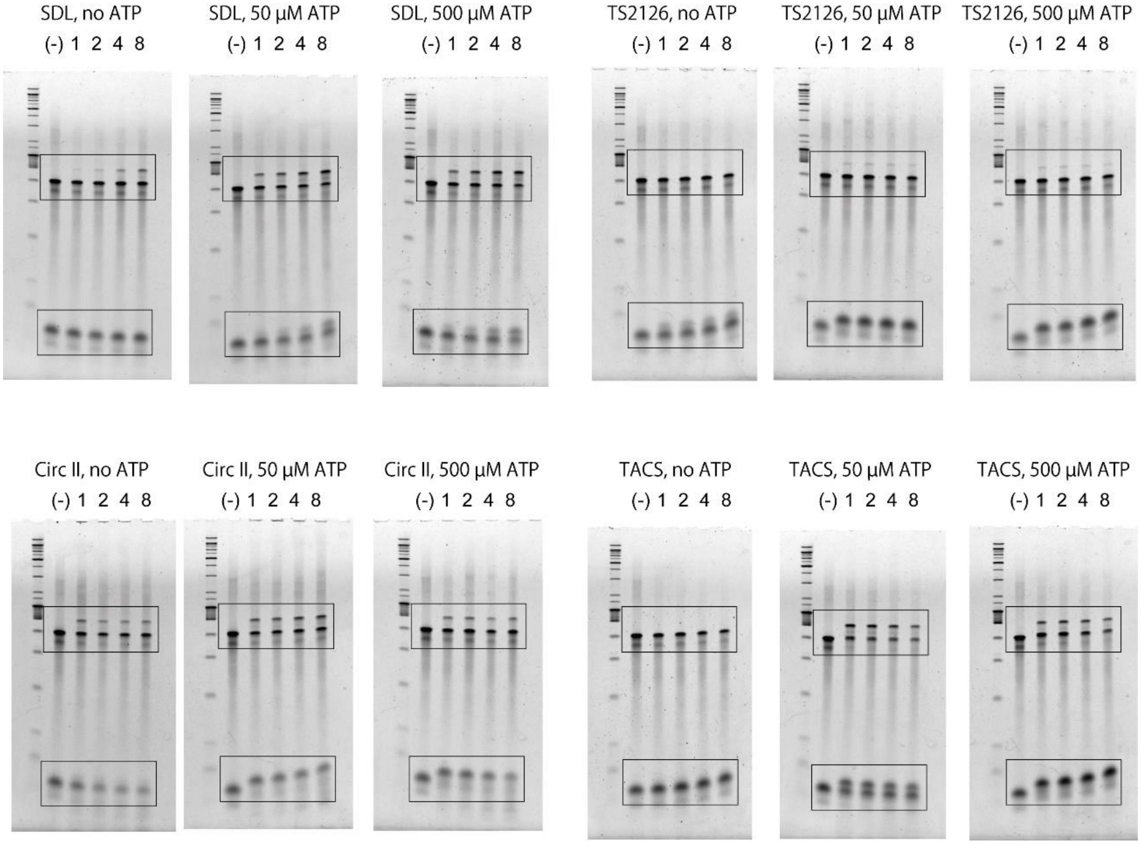

Figure 3A

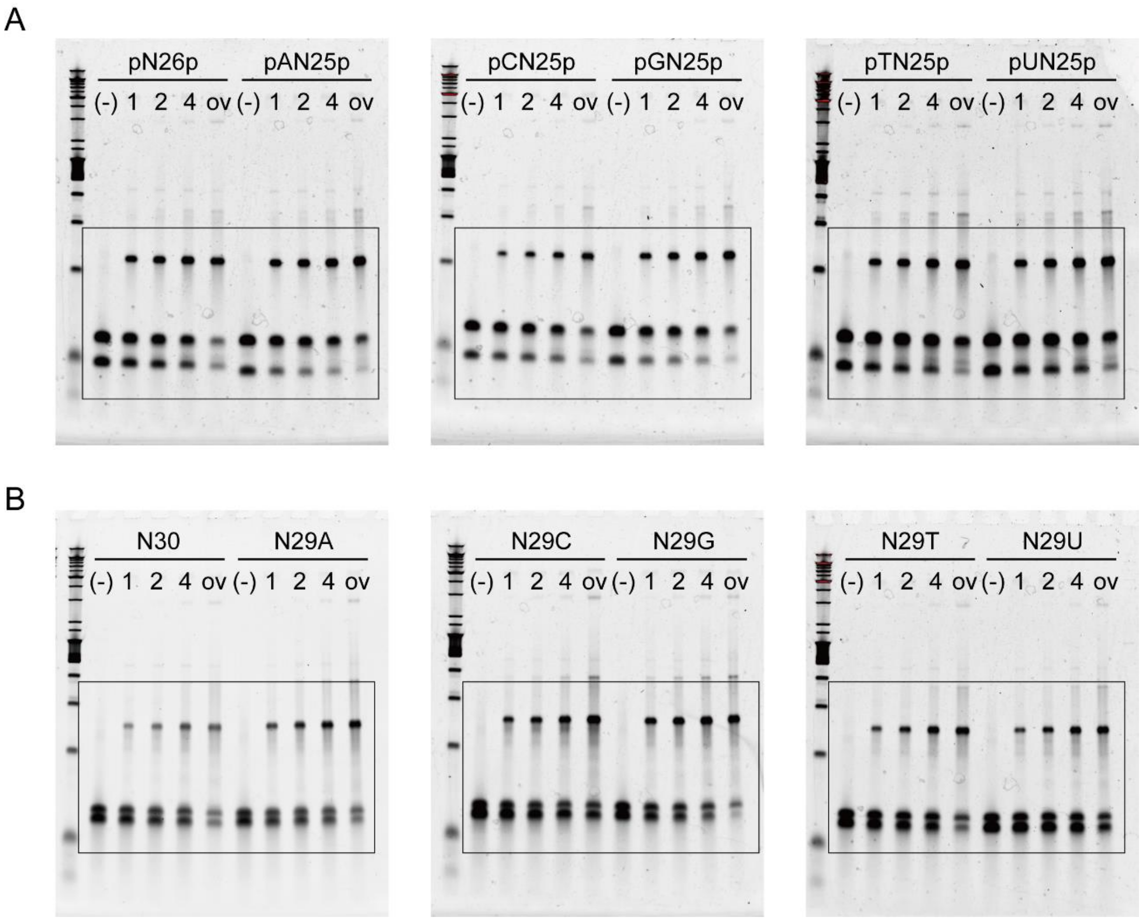
A
pN26p
pAN25p
(-) 1 2 4 ov (-) 1 2 4 ov
pCN25p
pGN25p
(-) 1 2 4 ov (-) 1 2 4 ov
pTN25p
pUN25p
(-) 1 2 4 ov (-) 1 2 4 ov
B
N30
N29A
(-) 1 2 4 ov (-) 1 2 4 ov
N29C
N29G
(-) 1 2 4 ov (-) 1 2 4 ov
N29T
N29U
(-) 1 2 4 ov (-) 1 2 4 ov
